# Ruthenium(II)–Arene Complexes with a 2,2′-Bipyridine Ligand as Anti-Aβ Agents

**DOI:** 10.3390/biom15040475

**Published:** 2025-03-25

**Authors:** Ryan M. Hacker, Jacob J. Smith, David C. Platt, William W. Brennessel, Marjorie A. Jones, Michael I. Webb

**Affiliations:** 1 Department of Chemistry and Biochemistry, SUNY Geneseo, Geneseo, NY 14454, USA; rh24@geneseo.edu (R.M.H.); jjs101@geneseo.edu (J.J.S.); 2 Department of Chemistry, Illinois State University, Normal, IL 69701, USA; dcplatt26@gmail.com (D.C.P.); majone3@ilstu.edu (M.A.J.); 3 Department of Chemistry, University of Rochester, Rochester, NY 14627, USA; william.brennessel@rochester.edu

**Keywords:** ruthenium therapeutics, Alzheimer’s disease, amyloid-β, human serum albumin

## Abstract

Agents that target the amyloid-β (Aβ) peptide associated with Alzheimer’s disease have seen renewed interest following the clinical success of antibody therapeutics. Small molecules, specifically metal-based complexes, are excellent candidates for advancement, given their relative ease of preparation and modular scaffold. Herein, several ruthenium–arene complexes containing 2,2-bipyridine (bpy) ligands were prepared and evaluated for their respective ability to modulate the aggregation of Aβ. This was carried out using the three sequential methods of thioflavin T (ThT) fluorescence, dynamic ligand scattering (DLS), and transmission electron microscopy (TEM). Overall, it was observed that **RuBA**, the complex with a 4,4-diamino-2,2-bipyridine ligand, had the greatest impact on Aβ aggregation. Further evaluation of the complexes was performed to determine their relative affinity for serum albumin and biocompatibility towards two neuronal cell lines. Ultimately, **RuBA** outperformed the other Ru complexes, where the structure–activity relationship codified the importance of the amino groups on the bpy for anti-Aβ activity.

## 1. Introduction

Alzheimer’s disease (AD) is a devastating neurological disorder that is characterized by extracellular deposits, the primary constituent of which is the peptide amyloid-beta (Aβ) [1]. Prepared by the enzymatic cleavage of the transmembrane amyloid precursor protein [2], Aβ is 40–42 amino acids long and central to the amyloid cascade hypothesis, where the self-assembly of the peptide in solution results in the formation of Aβ plaques within the brains of AD patients [3]. Within these plaques, endogenous metal ions, such as Fe, Cu, and Zn, are observed at elevated concentrations relative to nearby healthy tissue [4]. The coordination of the metals to Aβ has been observed to occur via histidine residues, of which there are three in Aβ (His-6, His-13, and His-14) [5]. Given the affinity of the peptide for metal ions, this spurred the development of metallotherapeutics that exploited this affinity, thereby forestalling its aggregation and the resultant cytotoxicity [6].

**Figure 1:**
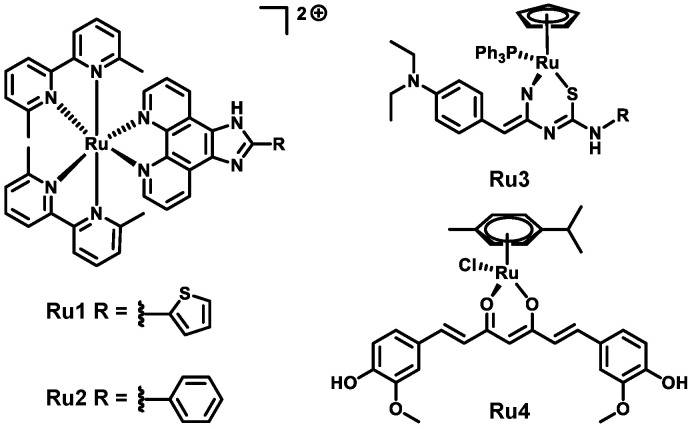
Previous Ru(II) complexes that have shown anti-Aβ activity.

Soluble oligomers of Aβ have a strong correlation with the progression of AD in contrast to deposited plaques [7], making them an important therapeutic target [8]. The use of small molecules, particularly metal complexes, to target Aβ has seen renewed interest, following the recently FDA-approved antibodies that target soluble Aβ in solution [9]. The unique advantages of metal complexes include a modular scaffold that can accommodate a variety of functional groups and molecular geometries that are inaccessible to organic compounds. In particular, ruthenium complexes have shown activity in modulating both the aggregation and cytotoxicity of Aβ in vitro. Structure–activity relationships (SARs) for these complexes identified the importance of a primary amine on the coordinated heterocyclic ligand eliciting anti-Aβ activity, which was accredited to hydrogen-bonding interactions with the peptide [10,11,12]. For such complexes, only a single heterocyclic group was attached to the metal center, while the use of ruthenium complexes containing bidentate chelating ligands has been dominated by coordinatively saturated metal centers [13], several of which have been used to detect Aβ_40_ fibrils in solution [14,15]. A recent report on the sterically strained Ru complexes **Ru1** and **Ru2** (Figure 1) saw photoactivation lead to the dissociation of a 6,6′-dimethyl-2,2-bipyridine ligand, which facilitated coordination to Aβ, thereby preventing its aggregation [16]. Alternatively, Ru–arene “piano stool” complexes with a single bidentate ligand have shown promise in modulating Aβ aggregation [17]. In particular, **Ru3** (Figure 1) was able to rescue *Caenorhabditis elegans* from Aβ_42_-induced paralysis [18], while the Ru–arene–curcumin complex **Ru4** (Figure 1) successfully limited the aggregation and cytotoxicity of Aβ_42_ [19].

**Figure 2:**
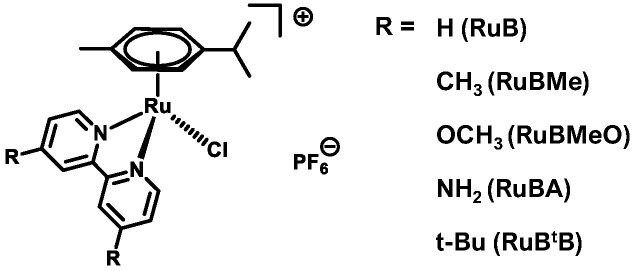
The Ru(II)–arene complexes prepared and evaluated for their respective anti-Aβ activity.

As a ligand, 2,2-bipyridine (bpy) and its derivatives have seen widespread usage in inorganic chemistry [20]; however, its investigation as a ligand for anti-Aβ complexes has seen success with both single ruthenium [21] and bimetallic complexes [22]. To determine the impact of the substitution at the 4′ position of the bpy ligand, a small series of complexes were prepared and evaluated for their respective anti-Aβ abilities (Figure 2). By expanding the utility of bpy ligands to Ru complexes that retain a labile chloride ligand, the activation of these complexes via external intervention is not anticipated. Furthermore, through the inclusion of several functional groups, SARs will be established, building upon those from previous anti-Aβ agents.

## 2. Materials and Methods

### 2.1. Materials

All chemicals were purchased from vendors unless specified otherwise. Chemicals used for synthesis and biological assays were obtained from Ambeed (2,2′-bipyridine, 4,4′-diamino-2,2′-bipyridine, 4,4′-dimethyl-2,2′-bipyridine, 4,4′-dimethoxy-2,2′-bipyridine, 4,4′-di-tert-butyl-2,2′-bipyridine), Oakwood Chemical (1,1,1,3,3,3,-hexafluoro-2-propanol (HFIP), ammonium hexafluorophosphate), Sigma-Aldrich (ruthenium(III) chloride hydrate), TCl America (dansyl glycine), and Thermo Fisher (alpha-terpinene, chloroform-D, deuterium oxide, dimethyl sulfoxide, methanol, methyl sulfoxide-D_6_, hexanes, diethyl ether). Human serum albumin was purchased from Sigma Aldrich as a lyophilized powder. Aβ_40_ was purchased from GenScript.

### 2.2. Instrumental Methods

An elemental analysis (EA) was collected at the University of Rochester’s Center for Enabling New Technologies Through Catalysis using a PerkinElmer 2400 Series II Analyzer. All NMR spectra were obtained using a Varian 400-MR 400 MHz NMR spectrometer. Diffraction data of single crystals were obtained using a Rigaku XtaLAB Synergy-S Dualflex diffractometer equipped with a HyPix-6000HE HPC area detector for data collection at 100 K. The full data collection was carried out using a PhotonJet (Cu) X-ray source. The structure was solved using SHELXT [23] and refined using SHELXL [24]. Most or all non-hydrogen atoms were assigned from the solution. Full-matrix least squares/difference Fourier cycles were performed, which located any remaining non-hydrogen atoms. All non-hydrogen atoms were refined with anisotropic displacement parameters. The N-H hydrogen atoms were found from the difference Fourier map and refined freely. All other hydrogen atoms were placed in ideal positions and refined as riding atoms with relative isotropic displacement parameters. See Tables S1 and S2 for additional crystal and refinement information.

### 2.3. Synthesis Procedures

#### 2.3.1. Ru–Arene Dimer

The Ru(II)–arene dimer ([Ru(η^6^-*p*-cymene)Cl_2_]_2_) was prepared following a previous procedure [25].

#### 2.3.2. General Procedure for the Ru Complexes

The synthesis of the Ru complexes followed the general procedure: the Ru dimer (0.1000 g, 0.163 mmol) and a bipyridine derivative (0.327 mmol) were dissolved in methanol (12.5 mL) and stirred for 1 h at room temperature. Next, ammonium hexafluorophosphate (0.490 mmol) was dissolved in methanol (5 mL) and was added to the solution dropwise, and the mixture was stirred for another hour at room temperature. The resulting solid was collected by vacuum filtration and washed with diethyl ether, then placed on a high vacuum line for approximately 6 h. For **RuBA**, no solid was observed after the initial mixing; however, after storage at −20 °C, a solid was formed and isolated as described previously.

#### 2.3.3. **RuB** ([Ru(η^6^-*p*-cymene)(2,2′-bipyridine)Cl]PF_6_)

Marigold-yellow powder (0.1060 g, 56.1% yield). ^1^H NMR (400 MHz, DMSO-D_6_, ppm): 9.50 (2H, dd), 8.61 (2H, d), 8.26 (2H, dt), 7.76 (2H, dt), 6.19, (2H, d), 5.95 (2H, d), 2.54 (1H, sept), 2.15 (3H, s), 0.91 (6H, d). ^13^C NMR (400 MHz, DMSO-D_6_, ppm): 18.73, 22.08, 30.82, 84.34, 87.12, 104.08, 124.22, 128.00, 140.36, 154.79, 156.25. EA results for C_20_H_22_N_2_ClRuPF_6_ theoretical: 42.00 C, 3.88 H, 4.90 N. Experimental: 41.96 C, 3.72 H, 4.83 N.

#### 2.3.4. **RuBMe** ([Ru(η^6^-*p*-cymene)(4,4′-dimethyl-2,2′-bipyridine)Cl]PF_6_)

Yellow-orange powder (0.0720 g, 37.9% yield). ^1^H NMR (400 MHz, DMSO-D_6_, ppm): 9.31 (2H, d), 8.46 (2H, s), 7.59 (2H, m), 6.14 (2H, d), 5.90 (2H, d), 2.14 (3H, s), 0.90 (6H, d). ^13^C NMR (400 MHz, DMSO-D_6_, ppm): 18.78, 21.08, 22.10, 30.81, 83.93, 86.85, 103.46, 103.83, 124.67, 128.56, 152.35, 154.43, 155.37. EA results for C_22_H_26_N_2_ClRuPF_6_ theoretical: 44.04 C, 4.37 H, 4.67 N. Experimental: 43.78 C, 4.24 H, 4.58 N.

#### 2.3.5. **RuBMeO** ([Ru(η^6^-*p*-cymene)(4,4′-dimethoxy-2,2′-bipyridine)Cl]PF_6_)

Yellow-orange powder (0.1462 g, 70.8% yield). ^1^H NMR (400 MHz, DMSO-D_6_, ppm): 9.23 (2H, d), 8.20 (2H, d), 7.31 (2H, dd), 6.09 (2H, d), 5.85 (2H, d), 4.02 (6H, s), 2.52 (1H, sept), 2.14 (3H, s), 0.92 (1H, d). ^13^C NMR (400 MHz, DMSO-D_6_, ppm): 18.79, 22.13, 30.81, 57.54, 83.39, 86.41, 102.97, 103.36, 110.73, 114.04, 156.28, 156.80, 168.17. EA results for C_22_H_26_N_2_O_2_ClRuPF_6_ theoretical: 41.81 C, 4.15 H, 4.43 N. Experimental: 41.85 C, 3.99 H, 4.53 N.

#### 2.3.6. **RuBA** ([Ru(η^6^-*p*-cymene)(4,4′-diamino-2,2′-bipyridine)Cl]PF_6_)

Earthy reddish-brown powder (0.1341 g, 67.5% yield). ^1^H NMR (400 MHz, DMSO-D_6_, ppm): 8.67 (2H, d), 7.04 (4H, d), 6.65 (2H, dd), 5.89 (2H, d), 5.65 (2H, d), 2.10 (3H, s), 0.92 (6H, d). ^13^C NMR (400 MHz, DMSO-D_6_, ppm): 18.78, 22.11, 30.76, 82.74, 85.76, 101.36, 102.22, 106.21, 111.44, 154.58, 155.01, 156.64. EA results for C_20_H_24_N_4_ClRuPF_6_ theoretical: 39.91 C, 4.02 H, 9.31 N. Experimental: 40.25 C, 4.39 H, 9.96 N.

#### 2.3.7. **RuBtB** ([Ru(η^6^-*p*-cymene)(4,4′-di-tert-butyl-2,2′-bipyridine)Cl]PF_6_)

Pastel yellow powder (0.0840 g, 37.4% yield). ^1^H NMR (400 MHz, CDCl_3_, ppm): 9.16 (2H, d), 7.96 (2H, d), 7.65 (2H, dd), 5.86 (2H, d), 5.68 (2H, d), 2.72 (1H, sept), 2.18 (3H, s), 1.41 (18H, s) 1.10 (6H, d). ^13^C NMR (400 MHz, CDCl_3_, ppm): 18.59, 21.99, 30.28, 31.11, 35.62, 84.78, 86.27, 102.17, 105.62, 119.63, 125.48, 154.31, 154.90, 164.49. EA results for C_28_H_38_N_2_ClRuPF_6_ theoretical: 49.16 C, 5.60 H, 4.09 N. Experimental: 48.97 C, 5.58 H, 4.13 N.

### 2.4. Log D

A stock solution was prepared from each Ru complex using dimethyl sulfoxide (DMSO), then diluted to 6 mL and a concentration of 50 μM using phosphate-buffered saline (PBS, pH 7.4), where the final DMSO concentration was less than 5%. The absorbance spectra were measured, then 6 mL of 1-octanol was added, and the resulting biphasic sample was mixed at room temperature for 2 h using an IKA Trayster inversion mixer (60 rpm). Following this, the aqueous layer was extracted, and its absorbance spectra were measured. Log D values were determined using the equation below:

$${LogD}_{7.4}=\log\left(\frac{Abs\;@\;\lambda_{max}\;before\;mixing}{Abs\;@\;\lambda_{max}\;after\;mixing}-1\right)$$

### 2.5. Thioflavin T Fluorescence Assay

All PBS used in the assay was filtered through a 0.20 μm Titan 3 syringe filter prior to use. The Ru stock solutions initially dissolved about 1.5 mg of each Ru complex in 100 µL DMSO and diluted it to 10 mL with PBS. Thioflavin T (ThT) was prepared in the same manner as the Ru complexes but was filtered prior to use in the assay. Lyophilized Aβ_40_ was monomerized following previous procedures [26] and stored at −20 °C until use. When removed from the freezer, peptide samples were held on ice for 20 min prior to preparation for the assay. The Aβ_40_ was mixed with a 15 μL aliquot of DMSO and gently flicked to initiate dissolution. Next, 135 μL PBS was added, and the sample was sonicated until the solution was clear. The concentration of the Aβ_40_ stock solution was determined using an Implen NanoPhotometer N50 at λ = 280 nm with an extinction coefficient of 1490 M^−1^ cm^−1^ [27]. Samples were prepared in a 96-well plate by the sequential addition of PBS, Ru, ThT, and Aβ, where the final volume was 150 μL, and the analyte concentrations were 10 μM each. Thioflavin T (ThT) fluorescence was measured using the SpectraMax M3 plate reader with λ_ex_ = 440 nm and λ_em_ = 480 nm. Measurements were taken every 30 min for a total of 6 h, with shaking occurring between each reading.

### 2.6. DLS Sample Preparation

Samples were extracted from the ThT assay plate for DLS analysis. A 125 μL sample was placed into a 1 mL syringe and injected through a syringe filter (pore size: 0.2 μm), into a 0.5 mL microcentrifuge tube. The filtrate was added to a microvolume polystyrene cuvette and placed into a Malvern Zetasizer Nano ZSP to measure the DLS spectra. Measurements are represented as an average of percent intensity through several runs determined by Zetasizer software Version 7.13.1.

### 2.7. TEM Sample Preparation

Samples for TEM were prepared using the DLS filtrates following their analysis. An aliquot (20 µL) was added directly to a copper-coated formvar/carbon 300 mesh grid with a 63 µm pore size (Ted Pella). After settling for 1 min, the solvent was wicked away, then the grid was stained using 2% uranyl acetate (20 µL). After standing for one minute, the solvent was wicked away, and the grid was washed using dH_2_O (20 µL), which stayed on the grid for one minute before being wicked away. The grids were stored at room temperature until analysis. TEM images were collected using a high-resolution JEOL JEM 2100F operating at 200 kV.

### 2.8. Protein Binding Assay

A stock of human serum albumin (HSA, 100 μM) was prepared using PBS, while the probes dansyl glycine (DG) and warfarin (WF) were prepared by initially dissolving them in DMSO and diluting them with PBS until the concentration of DMSO was less than 5%. Solutions of each Ru complex were prepared by initial dissolution in DMSO and subsequent dilution using PBS until the concentration of DMSO was less than 5%. The total volume of each sample was 3.5 mL, where the concentration of HSA and DG/WF was 2.5 μM throughout the experiment. The Ru concentration ranged from 0 μM to 62.5 μM, with an increase of 12.5 μM for each sample. In total, six samples were prepared for each protein binding assay. After preparation, the samples were placed on an IKA Trayster inversion mixer (60 rpm) for 30 s and incubated at approximately 28 °C in a Neslab gp-200 for 15 min, before being placed back on the inversion mixer (60 rpm) for 30 s. All fluorescence measurements for the protein binding assay were made using a PTI QuantiMaster 50, and the samples were measured at room temperature with conditions respective to the probe. DG had an excitation wavelength of 335 nm and an emission spectrum between 350 and 600 nm, with a step of 1 nm and integration of 0.2 s. WF maintained the same step and integration values but had an excitation wavelength of 295 nm and an emission spectrum between 330 and 500 nm.

### 2.9. Cell Viability Assays

Cultures of adherent *Rattus norvegicus* pheochromocytoma cells (PC-12; ATCC CRL-1721) and axenic *Rattus norvegicus* C6 glioma cells (ATTC CCL-107) were prepared as previously reported [28]. The cells were cultured using Dulbecco’s Modified Eagle’s medium with low glucose, sodium pyruvate, and without phenol red or L-glutamine (DMEM REF:11054-020; GIBCO; Waltham, MA, USA) and supplemented with 1.5% (*v*/*v*) heat-inactivated fetal bovine serum (GIBCO; Waltham, MA, USA), 10% (*v*/*v*) sterile filtered horse serum (Sigma-Aldrich, St. Louis, MO, USA), and 4% sterile L-glutamine (Sigma-Aldrich; St. Louis, MO, USA). Medium supplemented with sera was referred to as “complete medium”, while non-supplemented medium was designated as “incomplete”.

For the viability assays, both cell types were grown to confluency before being trypsinized, resuspended in 1 mL of complete medium per confluent well of a 6-well plate, diluted ten-fold, and then counted with a Scepter 2.0 Handheld Automated Cell Counter (Millipore, Burlington, MA, USA) affixed with a 60 µm sensor. Cells were subsequently seeded (adding 100 µL per well, thus adding an average of 1.0 × 10^4^ cells per well) into poly-L-lysine-coated flat-bottomed 96-wells using separate plates for each cell type. Stock solutions of the Ru complexes were prepared in DMSO, with an appropriate dilution using DMSO to the testing concentrations of 40, 20, 2, or 0.2 µM. In all cases, the final concentration of DMSO was 1% with cell incubations. Solutions of the Ru complexes or DMSO were introduced 24 h after cell plating and incubated a further 24 h unless otherwise indicated. Replicate wells (n = 4) were set up for each experimental variation. No cell controls were also set up, containing the Ru complex in DMSO additions, so that potential spectral signals could be blanked out for the spectrophotometric 3-[4,5-dimethylthiazol-2-yl]-2,5-diphenyltetrazoliumbromide (MTT) viability assay, as described below.

To assess the viability of the cells with and without Ru additions, cell viability tests were performed using an MTT assay following the procedure of Mosmann [29] with the following variations: Before adding the MTT reagent (5 mg/mL water), 90 µL of the complete medium was removed from each well, and 90 µL of incomplete medium was added. Then, 10 µL of MTT reagent was added per well, gently mixed, and incubated at ambient temperature for one hour. The reactions were stopped by the addition of 100 µL per well of the stopping reagent (10% *v*/*v* Triton X-100, 1 mM HCl in isopropanol) to lyse the cells and dissolve the formed formazan crystals. The absorbance values were recorded using a Bio-Rad^®^ Microplate Reader Benchmark at 595 nm. All MTT data were normalized to the DMSO controls by calculating the average percent of DMSO control with the following formula:

$$\frac{average\;\left(cell+Ru\;MTT\right)-average\;(Ru\;blank\;MTT)}{average\;\left(control\;cell\;MTT\right)-average\;(control\;blank\;MTT)}\;\times \;100$$

Data were obtained from 4 replicates and reported as a percent of control cells.

## 3. Results and Discussion

### 3.1. Synthesis of the Ru Complexes

**Figure 3:**
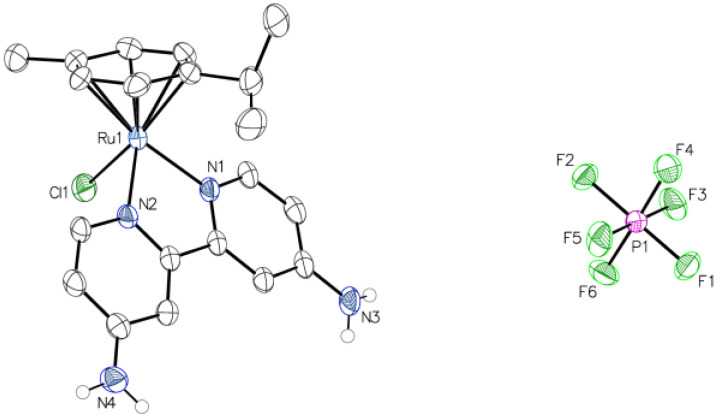
X-ray crystal structure of **RuBA**. The ellipsoids of all non-hydrogen atoms are shown at the 50% probability level.

Preparation of the Ru complexes was achieved following the mixing of a Ru(II)–arene dimer with the desired 2,2-bipyridne (bpy) ligands. The subsequent addition of ammonium hexafluorophosphate afforded the desired complex ions. The hexafluorophosphate salts of **RuB**, **RuBMe**, and **RuBtB** have been reported previously [30,31,32], along with the chloride salts of **RuBMeO** and **RuBA** [33,34]. To confirm successful preparation of the complexes, an analysis by ^1^H and ^13^C NMR was performed, while yields similar to those of the previous reports were obtained. Fortunately, single crystals suitable for X-ray diffraction were obtained for **RuBA,** and its structure was solved (Figure 3). The observed molecular structure was the anticipated three-legged “piano stool” arrangement with a pseudo-octahedral geometry. The measured bond lengths to the facial ligands around the Ru metal center of Ru-Cl 2.409(9) Å, along with the bpy Ru-N 2.085(3) Å and 2.091(3) Å, were in good agreement with those of **RuB** and **RuBMeO** [33,35]. Hydrogen bonding interactions were observed between the free amine groups and the hexafluorophosphate anion within the unit cell (Table S2). Since studies of previous Ru-based anti-Aβ complexes identified a free amine group on the heterocyclic ligand as an important functional group for activity [10,11,12], the observation of such hydrogen-bonding interactions within the crystal structure was encouraging.

### 3.2. Log P

The ability of Aβ-targeting therapeutics to cross the blood–brain barrier (BBB) is essential for their success. The BBB is a highly selective lipophilic membrane that plays an important role in the protection of the brain from both exogenous and endogenous toxins, where passage across this membrane is a common point of failure for central nervous system drugs [36]. For predicting passage across the BBB, one metric that is used is the n-octanol/water partition coefficient of the molecule (log P) [37]. For Ru therapeutics, pH can play an important role in the speciation and stability of the complex [38,39]; therefore, the determination of log D was performed. For such an analysis, the complexes were initially dissolved in DMSO, then diluted using PBS. After initial absorption measurements, the aqueous samples were mixed with an equal volume of 1-octanol for 2 h. The short mixing time is necessary to minimize any ligand exchange that may occur (vide infra), while still providing an accurate value for the partitioning [11]. Overall, four of the five compounds evaluated had negative log D_7.4_ values, indicating that the complexes are hydrophilic (Table 1). The most hydrophilic was **RuB**, while the other complexes had log D_7.4_ values from −1.0 to +1.0, steadily increasing thus: **RuBMe** < **RuBMeO** < **RuBA** < **RuBtB** (Table 1). To the best of our knowledge, the partitioning for these complexes has not been evaluated previously, while the values obtained are similar to previous Ru(II)–arene complexes with bidentate ligands [40].

### 3.3. Aqueous Stability

A common step in the mechanism of action for most metal-based drugs is ligand exchange around the metal center. This promotes the coordination of the complex to the penultimate biological target, eliciting the desired response [41]. For ruthenium therapeutics, such an exchange has been observed to readily occur under physiological conditions [39]. Therefore, to evaluate the relative stability of the prepared complexes, ^1^H NMR studies were performed. Following complete dissolution of the complexes in deuterated DMSO, dilution using D_2_O to a final DMSO concentration of 25% provided aqueous samples that were measured following prolonged incubation at 37 °C. At DMSO levels below 25%, immediate precipitation occurred for all five complexes, while for **RuBtB**, 75% DMSO was necessary to maintain solubility.

For the majority of the complexes, new signals emerged in close proximity to those of the parent complex, even without any incubation. Surprisingly, these new signals reached a maximum intensity after only 1 h of incubation for **RuB**, **RuBMe**, and **RuBMeO**, while an extended incubation up to 24 h yielded no substantial differences in the spectra. By contrast, for **RuBA**, the initial spectrum with no incubation was nearly indistinguishable from that after 24 h of incubation, indicating that ligand exchange for this complex was particularly rapid. The only complex that did not show any new signals within the initial spectrum was **RuBtB**. In fact, **RuBtB** was the only complex for which no new signals emerged even after 24 h of incubation, a likely consequence of the low amount of D_2_O in the sample (25%). A previously study monitoring ligand exchange for **RuB** in the presence of several proteins saw that neither the arene ring nor the 2,2-bipyridine ligands were lost, but rather the labile monodentate chloride ligand was replaced [42]. A similar phenomenon is anticipated for the prepared complexes, as the new signals which emerged in the NMR spectra did not match those of the free ligands.

**Figure 4:**
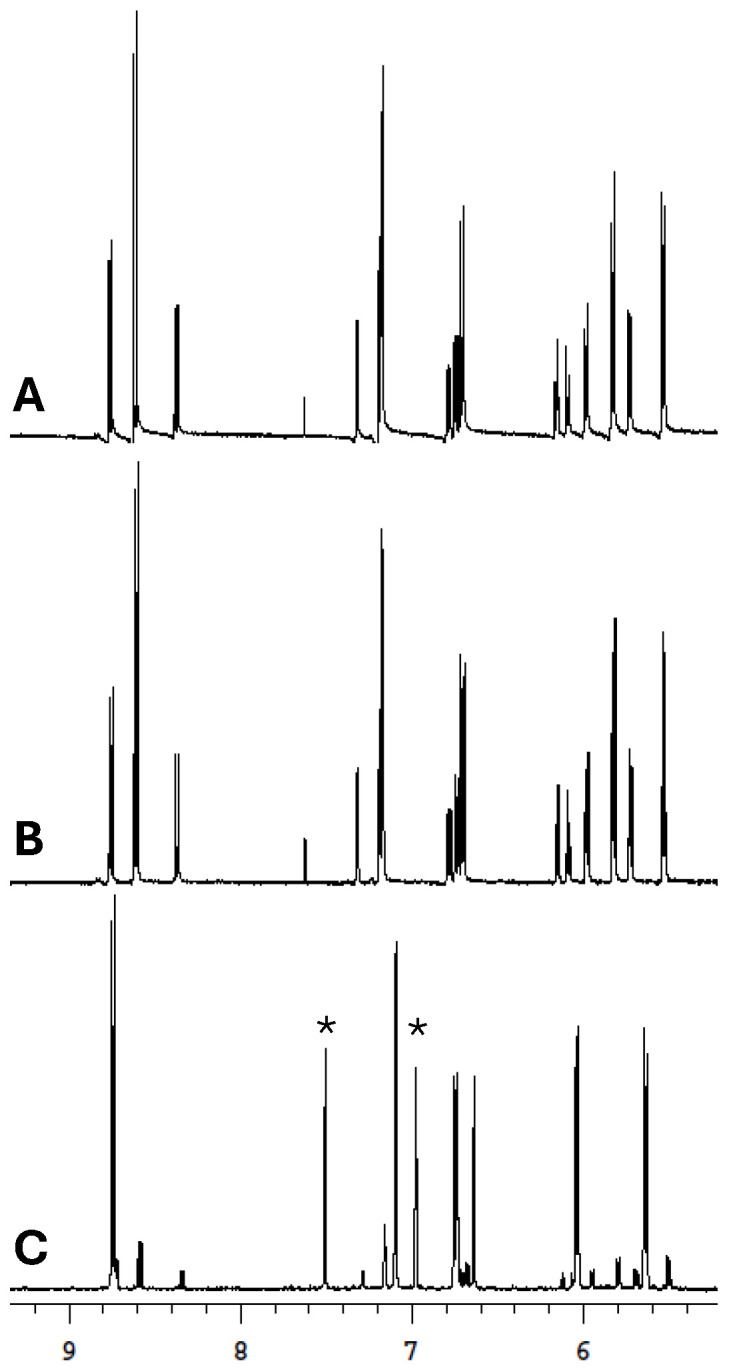
^1^H NMR spectra in 25% DMSO-D_6_ and D_2_O of complex **RuBA**. (**A**) **RuBA** with no incubation, (**B**) **RuBA** after 1 h of incubation at 37 °C, (**C**) imidazole and **RuBA** (1:1 molar ratio) after 1 h of incubation at 37 °C. The free imidazole peaks are marked with an asterisk (*).

Coordination of metal-based therapeutics to the Aβ peptide has been observed to occur primarily via the histidine side chains at positions His-6, His 13, and/or His-14 [43,44,45]. To evaluate the likelihood of such a coordination occurring for the prepared Ru complexes, they were each mixed with an equimolar amount of imidazole, followed by extended incubation at 37 °C. For most of the compounds, immediately following dissolution new features were observed within the ^1^H NMR spectrum. These signals were in close proximity to those of the arene ring and bpy ligand, while remaining distinct from the sample in deuterated solvent alone (Figures S16–S19). Following 1 h of incubation, the intensity of these signals increased, further supporting their assignment as new features, which are attributed to imidazole coordination. This is best exemplified with the complex **RuBA** (Figure 4), where the new signals within the aromatic region of the NMR spectrum following incubation with imidazole do not match with those for samples in aqueous media alone. Unsurprisingly, the lone complex for which no changes were observed was **RuBtB**. Given the low water solubility of the complex, samples were made in pure CDCl_3_ with imidazole, where no changes in the spectra were observed for the duration of the experiment (Figure S20). Taken together, the majority of the prepared complexes displayed some association with imidazole in solution, thereby suggesting that interactions with the Aβ peptide could proceed via the histidine imidazole side chains.

### 3.4. ThT Assay

To determine the relative impact of each complex on the aggregation of Aβ, an assay was completed using the fluorometric probe Thioflavin T (ThT). In the presence of aggregates of Aβ, following excitation around 450 nm, ThT undergoes a characteristic emission of light around 485 nm [46]. This phenomenon is sensitive to interactions with Aβ deposits, as the free rotation of the benzothiazole ring is inhibited, thereby leading to selective turn-on fluorescence, which has been shown to correlate to the aggregation of the peptide [47]. For anti-Aβ therapeutics, this is a common first line of evaluation for the ability to mitigate this aggregation, while also allowing for comparisons between treatments, allowing for the determination of structure–activity relationships (SARs).

**Figure 5:**
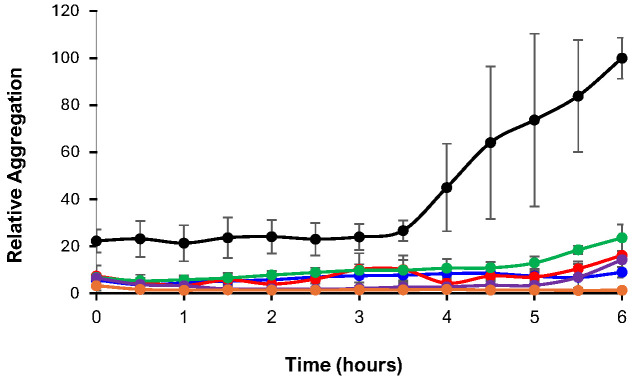
ThT fluorescence results following the incubation of equimolar solutions (10 μM) of Aβ_40_ with the Ru complexes for up to 6 h at 37 °C. The signals were normalized to the positive control of the free peptide in solution at the 6 h time point. The error bars represent the standard deviation observed for each data point for the respective samples. Each sample was measured in triplicate, where Black = Aβ_40_ Alone; Blue = Aβ_40_ + **RuB**; Green = Aβ_40_ + **RuBMe**; Red = Aβ_40_ + **RuBMeO**; Orange = Aβ_40_ + **RuBA**; Purple = Aβ_40_ + **RuBtB**.

Each of the prepared Ru complexes was mixed with an equimolar amount of Aβ_40_ (10 µM), along with ThT (10 µM). Each sample was prepared in triplicate, loaded onto a microwell plate, and incubated at 37 °C with constant agitation to promote aggregation, with fluorescence measurements taken every 30 min for up to 6 h. The resultant fluorescence signals were normalized to the last time point for the samples of Aβ_40_ alone. After 4 h of incubation, a sharp increase in fluorescence for the peptide alone was observed, signifying that aggregation was occurring (Figure 5). This was in stark contrast to the peptide following treatment with the respective Ru complexes, as a minimal increase in fluorescence was observed for the duration of the experiment (Figure 5). This was most prominent for **RuBA**, where virtually no increase was observed for the duration of the experiment, suggesting that this complex was very effective in limiting Aβ_40_ aggregation. Using the final 6 h time point, the relative aggregation of each Aβ_40_ by each complex increased in the following order: **RuBMe** (23.6% ± 5.7%), **RuBMeO** (16.1% ± 6.9%), **RuBtB** (14.3% ± 0.6%), **RuB** (9.0% ± 2.3%), and **RuBA** (1.3% ± 0.9%). Remarkably, the activity shown by **RuBA** is among the greatest we have measured thus far across a variety of Ru complexes. To confirm this activity, the samples from the ThT assay were subjected to additional analysis, as described below. 

### 3.5. DLS

Dynamic light scattering (DLS) was used to determine the relative sizes of the Aβ particles in solution. This non-invasive technique utilizes laser light to excite particles in solution; then, based upon their Brownian motion a size distribution profile is created. This has been used previously to monitor the aggregation of Aβ in solution, as the free peptide has a signal near 1 nm [48], while aggregates of the peptide have substantially larger, and variable, particle sizes [49].

**Figure 6:**
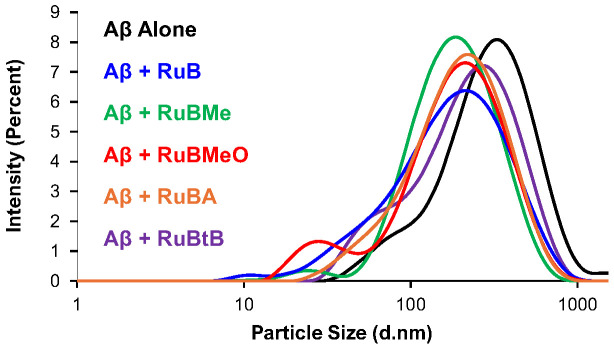
Particle size distributions (hydrodynamic radii, d. nm) of Aβ_40_ with and without co-incubation with Ru complexes for 6 h in PBS (pH 7.4, 37 °C), where Black = Aβ_40_ alone; Blue = Aβ_40_ + **RuB**; Green = Aβ_40_ + **RuBMe**; Red = Aβ_40_ + **RuBMeO**; Orange = Aβ_40_ + **RuBA**; Purple = Aβ_40_ + **RuBtB**.

Samples for DLS analysis were taken directly from the ThT assay following its completion. This allowed for a comparison between both data sets, to determine if a correlation for activity between the complexes could be established. For the Aβ_40_ peptide alone, a prominent peak around 340 nm was observed, while following treatment with the respective Ru complexes, a discernable shift to smaller particle sizes was unanimously observed (Figure 6). For these prominent peaks, the smallest particle sizes were observed for **RuBMe** (214 nm), followed by **RuB** (227 nm), **RuBA** (231 nm), **RuBMeO** (239 nm), and **RuBtB** (264 nm).

Surprisingly, in addition to the predominant peaks, a secondary feature was also observed in the distribution profiles for each sample. For Aβ_40_, this was more of a shoulder to the main peak, and it was observed at 78 nm, accounting for 13 percent of the total signal. For the peptide following treatment with the Ru complexes, this feature was a more discernable peak, which was shifted to substantially smaller particle sizes. For these minor features, the smallest particle size was observed for RuB at 21 nm, accounting for 4% of the total spectrum. This was followed by **RuBMe** (24 nm, 2%), **RuBMeO** (32 nm, 11%), **RuBA** (42 nm, 3%), and **RuBtB** (53 nm, 4%). As noted, these features represented anywhere from 2% to 11% of the entire spectrum; however, since DLS has a disproportional sensitivity to large particles over small particles [50], even a seemingly miniscule percentage is nonetheless important. Indeed, this bimodal distribution has been observed previously for Ru therapeutics [51] and is supportive of the anti-Aβ nature of the complexes. Taken together, using the peak sizes and relative percent for each signal, the quantitative ranking of the impact of the complexes on Aβ_40_ aggregation by DLS was determined to be **RuBMe** > **RuBMeO** > **RuB** > **RuBA** > **RuBtB**.

### 3.6. TEM

**Figure 7:**
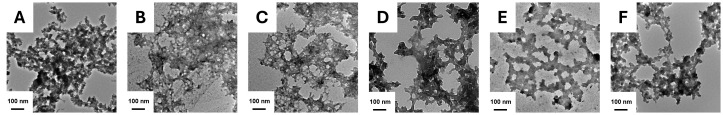
TEM images taken at 100 kX magnification of the DLS filtrates: (**A**) Aβ_40_ alone, (**B**) Aβ_40_ + **RuB**, (**C**) Aβ_40_ + **RuBMe**, (**D**) Aβ_40_ + **RuBMeO**, (**E**) Aβ_40_ + **RuBA**, (**F**) Aβ_40_ + **RuBtB**.

The final evaluative step in the Aβ_40_ aggregation assay was imaging the samples using transmission electron microscopy (TEM). The samples used in the preparation of the TEM grids were those of the DLS filtrates, thereby allowing for a direct comparison between experimental methods, ultimately resulting in the same sample being measured by three different instruments. In the absence of any Ru complexes, large, dense, amorphous aggregates were observed for Aβ_40_ (Figure 7A). When co-incubated with the respective Ru complexes, the observed amorphous aggregates of Aβ_40_ were notably disrupted, as the features observed were elongated and less dense than those of the peptide alone (Figure 7B–F). In order to determine the impact of each complex on Aβ_40_, multiple images were taken from each prepared grid (Figure S21), thereby providing an accurate representation of the particles. Using these images, the complex with the greatest anti-Aβ activity was **RuBA**, as images from this sample yielded the most dispersed and least dense particles. Overall, a qualitative ranking of the complexes was performed, where the anti-Aβ activity increased as follows: **RuBMeO** < **RuBtB** < **RuBMe** < **RuB** < **RuBA**.

### 3.7. HSA Binding

**Figure 8:**
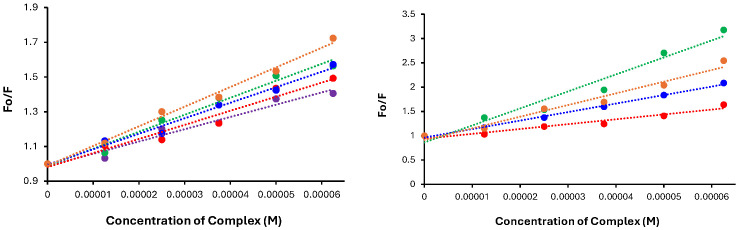
Stern–Volmer plots for the complexes after mixing them with HSA and DG (**left**) and WF (**right**). Blue = **RuB**, Green = **RuBMe**, Red = **RuBMeO**, Orange = **RuBA**, Purple = **RuBtB**.

One important consideration for a potential AD therapeutic is its interaction with serum proteins, specifically albumin (HSA). As the most abundant protein in blood, HSA is a known transporter of hydrophobic molecules, including several drugs such as ibuprofen, paclitaxel, and valproic acid [52,53,54]. Since HSA does not cross the BBB, potential neurotherapeutics would ideally have a low affinity for this protein. To determine the potential association of the prepared Ru complexes with HSA, a protein binding assay was performed. For these studies, the displacement of two fluorometric probes was used to determine the relative affinity of the Ru complexes for two of the established binding sites on HSA. The first probe, warfarin (WF), an anticoagulant, binds selectively to Sudlow site I on HSA [55], while the second probe used was dansyl glycine (DG), which selectively binds to Sudlow site II on HSA [56]. As these are the two major binding sites on the protein, the probes allow for observations of binding events that occur within these hydrophobic pockets. Individually, the probes were initially mixed with HSA, followed by increasing amounts of the respective Ru complexes, whereby the decrease in fluorescence signals from the respective probes (Figures S22–S30) was used to construct a Stern–Volmer plot, where the slope of the line represents the conditional binding constant (K’) for the respective Ru complexes to each site (Figure 8).

Beginning with WF displacement, the conditional binding constants to HSA ranged from 4.00 to 4.56. Unfortunately, background fluorescence for **RuBtB** was observed, making measurements with WF unfeasible. For the remaining complexes, the strongest binding was observed for **RuBMe**, followed by **RuBA**, **RuB**, and **RuBMeO**. Fortunately, background fluorescence from the complexes with DG was not an issue; therefore, the affinity of all five complexes for HSA could be determined. For this probe, the binding constants were all very close to one another, ranging from 3.85 to 4.07. The complex with the highest binding constant was **RuBA**, followed by **RuBMe**, **RuB**, **RuBMeO,** and **RuBtB**. In each case, binding site I was preferred for each Ru complex, which is not unexpected given the affinity of heterocyclic groups for this site [57]. Overall, the binding constants measured were of a similar magnitude to previously described anti-Aβ Ru-arene complexes [58], while they remained substantially lower than that of ibuprofen (log K = 6.30) [59] and indomethacine (log K = 5.31), a non-steroidal anti-inflammatory drug (NSAID) that can affect the brain by reducing intracranial pressure and improving cerebral perfusion [60].

### 3.8. Cell Viability Assay

**Figure 9:**
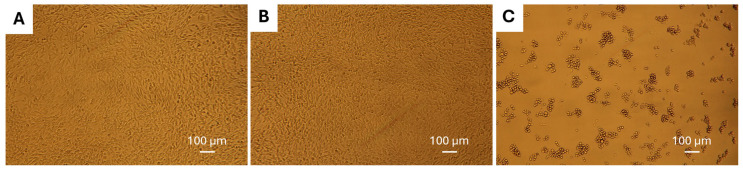
C6 cells following 24 h of incubation with (**A**) DMSO only, (**B**) 20 µM **RuBA**, and (**C**) 20 µM **RuBtB**.

A final step in the evaluation of the Ru complexes was completed using a standard MTT cell viability assay towards two neuronal cells lines. The cell lines used were glial cells, as these are the most abundant cell type within the brain [61], and chromaffin cells, which would provide an indication regarding stress response [62]. Taken together, these would provide information regarding the overall biocompatibility of the Ru complexes. Following 24 h of incubation with both cell types, the Ru complexes tested displayed low cytotoxicity at the concentrations evaluated (Figure S31). The lone exception was **RuBtB** at 20 and 40 µM, for which substantial toxicity was observed with both the C6 and P12 cells. This toxicity was evident as the morphology of the cells changed, as shown via images taken using confocal microscopy, where the toxic **RuBtB** is juxtaposed to the biocompatible **RuBA** (Figure 9). Both the DMSO-treated control cells and those with **RuBA** are virtually indistinguishable, while those with **RuBtB** are severely reduced in numbers and cell shapes. This discrepancy was also observed when comparing the cellular morphologies of **RuBtB** to the other Ru complexes for both cell lines (Figures S32 and S33).

## 4. Conclusions

Given the renewed interest in Aβ-targeting therapeutics, a small series of Ru(II)–arene complexes with variable bpy ligands were prepared and evaluated for their respective abilities to modulate the aggregation of the peptide. Specifically, SARs would be established for the various functional groups, thereby advancing the field of metallotherapeutics for AD. Initially, the aqueous stability of the complexes was determined using ^1^H NMR spectroscopy, where ligand exchange was observed for most of the complexes. To further evaluate if this phenomenon facilitates coordination to the Aβ peptide via histidine, the complexes were mixed with equimolar amounts of imidazole, and the NMR spectra were measured. New signals emerged for most of the complexes, signifying that coordination of the Ru metal center to imidazole had occurred.

Having established the likely association between the complexes and the peptide, their respective impact on its aggregation was evaluated using the sequential analysis of ThT fluorescence, DLS, and TEM imaging. Across all five complexes, consistent anti-Aβ activity was observed. Additionally, the complexes were evaluated for their ability to bind to HSA, since this is a known transporter of ruthenium therapeutics [63]. While all of the complexes displayed affinity for the serum protein, the measured binding constants were substantially lower than a previous Ru therapeutic [64], while being similar to other Ru–arene complexes [65]. Lastly, the biocompatibility of the complexes was evaluated against two neuronal cells lines. Only **RuBtB** was observed to have an IC_50_ below the concentrations evaluated, while the remaining complexes had IC_50_ values above those used in the assay, indicating that they were well tolerated by the cells.

To determine SARs, the performance of each complex was ranked relative to its peers for each experimental method from 1–5 (1 = best, 5 = worst). Using these rankings, average scores for each complex were determined (Table 2), where the different functional groups on the bpy ligand and their resultant impact on the performance of the complex were codified. Taken together, **RuBA** was identified as the complex having the greatest overall performance. This is in good agreement with previous Ru(III)-based anti-Aβ complexes [10,11,12], where the incorporation of a primary amine into the ligand backbone was observed to consistently yield the greatest anti-Aβ activity. Importantly, the anti-Aβ activity observed for **RuBA** is comparable to several of those Ru(III) predecessor complexes. This represents an exciting new direction for Ru(II)–arene complexes, as the bidentate bpy will undoubtedly spur the investigation into alternative ligands, in the quest for the ultimate anti-Aβ therapeutic.

## Figures and Tables

**Table 1:** The experimentally determined log D_7.4_ values for each Ru complex, along with the conditional binding constants to serum albumin determined using warfarin (WF) and dansyl glycine (DG).

	Log D_7.4_	Log *K* (DG)	Log *K* (WF)
**RuB**	−1.90 ± 0.15	3.96	4.24
**RuBMe**	−0.99 ± 0.13	3.99	4.56
**RuBMeO**	−0.74 ± 0.08	3.91	4.00
**RuBA**	−0.55 ± 0.03	4.07	4.38
**RuBtB**	0.84 ± 0.03	3.85	—

**Table 2:** Summary of the rankings of prepared Ru complexes for their ability to modulate the aggregation of Aβ_40_ (ThT fluorescence, DLS, TEM) and to bind to HSA and for their cytotoxicity towards C6 glioma cells, on a scale of 1 to 5 (1 = best, 5 = worst).

	ThT	DLS	TEM	HSA	Cytotoxicity	Average
**RuB**	2	3	2	3	4	2.8
**RuBMe**	5	1	3	4	2	3
**RuBMeO**	4	2	5	2	3	3.2
**RuBA**	1	4	1	5	1	2.4
**RuBtB**	3	5	4	1	5	3.6

## Data Availability

The data supporting this article have been included as part of the ESI. crystallographic data for **RuBA** deposited at the CCDC under 2420394.

## Supplementary Materials

The following supporting information can be downloaded at: https://www.mdpi.com/article/10.3390/biom15040475/s1, Figures S1–S10: ^1^H and ^13^C NMR spectra for the prepared Ru complexes. Figures S11–S15: ^1^H spectra for the prepared Ru complexes in 25–75% DMSO-D_6_ in D_2_O. Figures S16–S20: ^1^H spectra for the prepared Ru complexes with imidazole in either D_2_O/DMSO-D_6_ or CDCl_3_. Figure S21: Additional TEM images collected for all of the Ru complexes with Aβ_42_ from the DLS filtrates. Figures S22–S30: Fluorescence emission spectra at various Ru-HSA ratios by the titration of HSA-DG (1:1) or HSA-WF (1:1) with each Ru complex. Figure S31: Cell viability for C6 and P12 cells with the Ru complexes. Figure S32: Images of the C6 cells following incubation with the Ru complexes. Figure S33: Images of the P12 cells following incubation with the Ru complexes. Table S1: Refinement parameters for the X-ray crystal structure of **RuBA**. Table S2: Hydrogen bonding parameters within the crystal structure of **RuBA**.
