# Supplementary material for: Ruthenium(II)–Arene Complexes with a 2,2′-Bipyridine Ligand as Anti-Aβ Agents
Source: Biomolecules. 2025 Mar 25;15(4):475. doi: 10.3390/biom15040475 (PMC12024814; doi:10.3390/biom15040475)
Supplement: Supplementary file 1 [file biomolecules-15-00475-s001.zip › biomolecules-3531865-supplementary.pdf]

Supplementary Information For:

Ruthenium(II)-arene Complexes With a 2,2-Bipyridine Ligand as Anti-Aβ Agents

Ryan M. Hacker,[a] Jacob J. Smith,[a] David C. Platt,[b] William W. Brennessel,[c] Marjorie A. Jones,[b] Michael I. Webb[a,*]

1 Department of Chemistry and Biochemistry, SUNY Geneseo, Geneseo, NY, 14454

2 Department of Chemistry, Illinois State University, Normal, IL, 61790

3 Department of Chemistry, University of Rochester, Rochester, NY, 14627

Corresponding Author: Michael I. Webb, mwebb@geneseo.edu

Table of Contents:

- **Figures S1-S10:** ^{1}H and ^{13}C NMR spectra for the prepared Ru complexes.
- **Figures S11-S15:** ^{1}H spectra for the prepared Ru complexes in 25-75% DMSO-D_6 in D_2O.
- **Figures S16-S20:** ^{1}H spectra for the prepared Ru complexes with imidazole in either D_2O/DMSO-D_6 or $CDCl_3$.
- **Figure S21:** Additional TEM images collected for all of the Ru complexes with $A\beta_{42}$ from the DLS filtrates.
- **Figures S22-S30:** Fluorescence emission spectra at various Ru-HSA ratios by the titration of HSA-DG (1:1) or HSA-WF (1:1) with each Ru complex.
- **Figure S31:** Cell viability for C6 and P12 cells with the respective Ru complexes.
- **Figure S32:** Images of the C6 cells following incubation with the Ru complexes.
- **Figure S33:** Images of the P12 cells following incubation with the Ru complexes.
- **Table S1:** Refinement parameters for the X-ray crystal structure of **RuBA**.
- **Table S2:** Hydrogen bonding parameters within the crystal structure of **RuBA**.

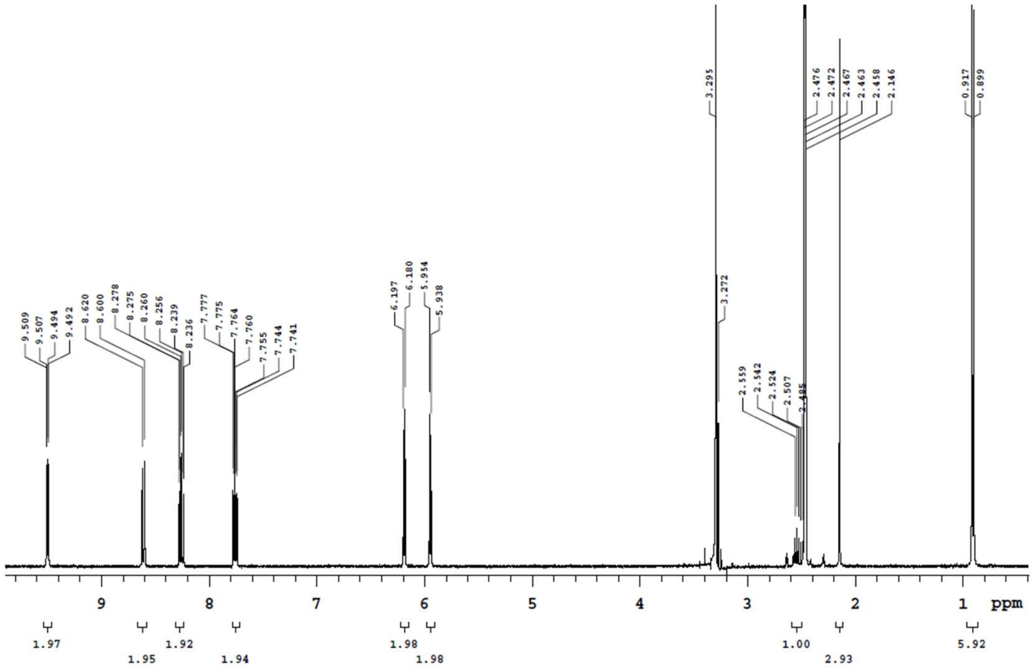

Figure S1. ^{1}H NMR spectrum of complex **RuB** in DMSO-D_6.

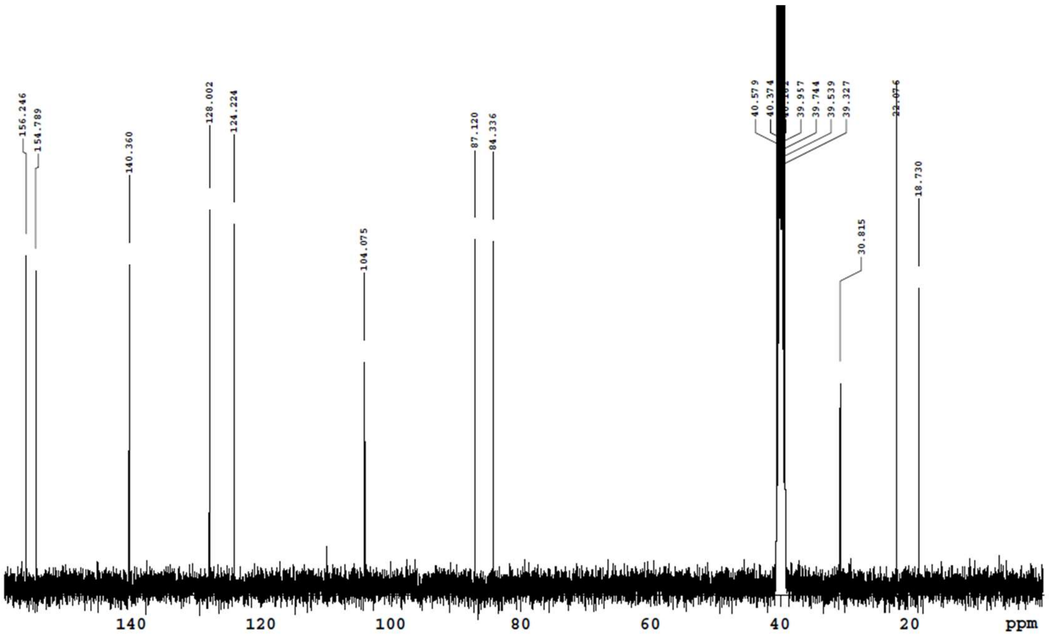

Figure S2. ^{13}C NMR spectrum of complex **RuB** in DMSO-D_6.

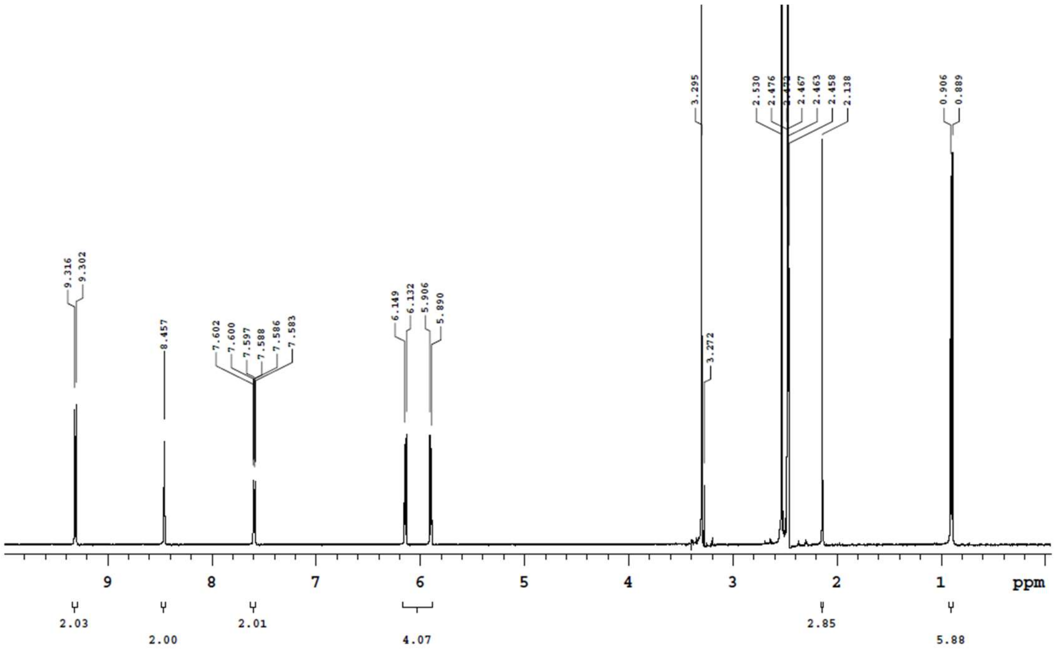

Figure S3. ^{1}H NMR spectrum of complex **RuBMe** in DMSO-D$_6$.

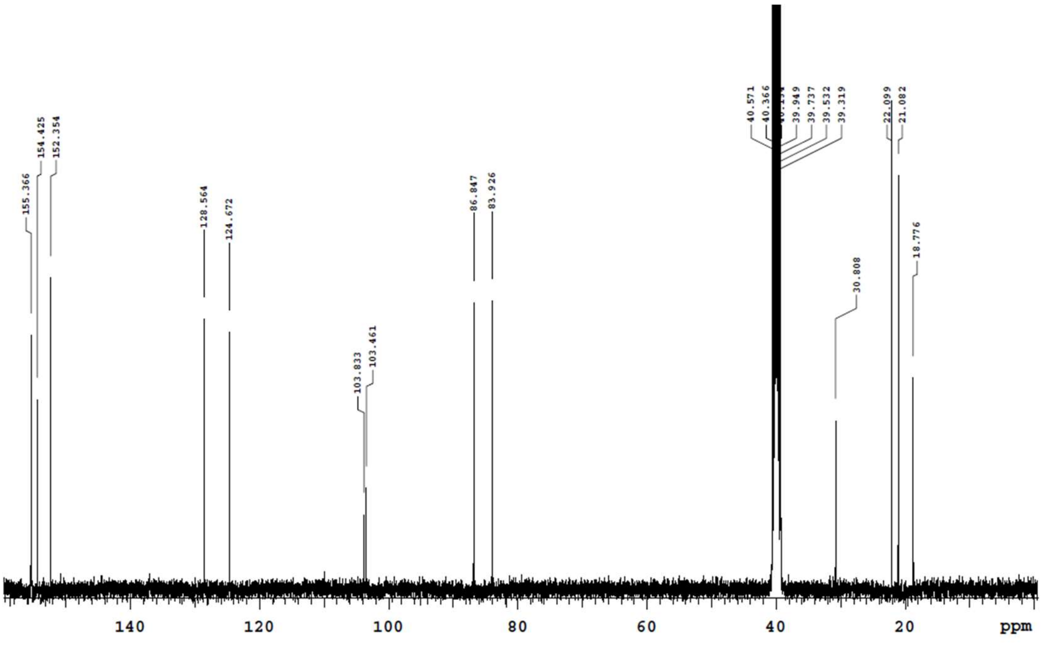

Figure S4. ^{13}C NMR spectrum of complex **RuBMe** in DMSO-D$_6$.

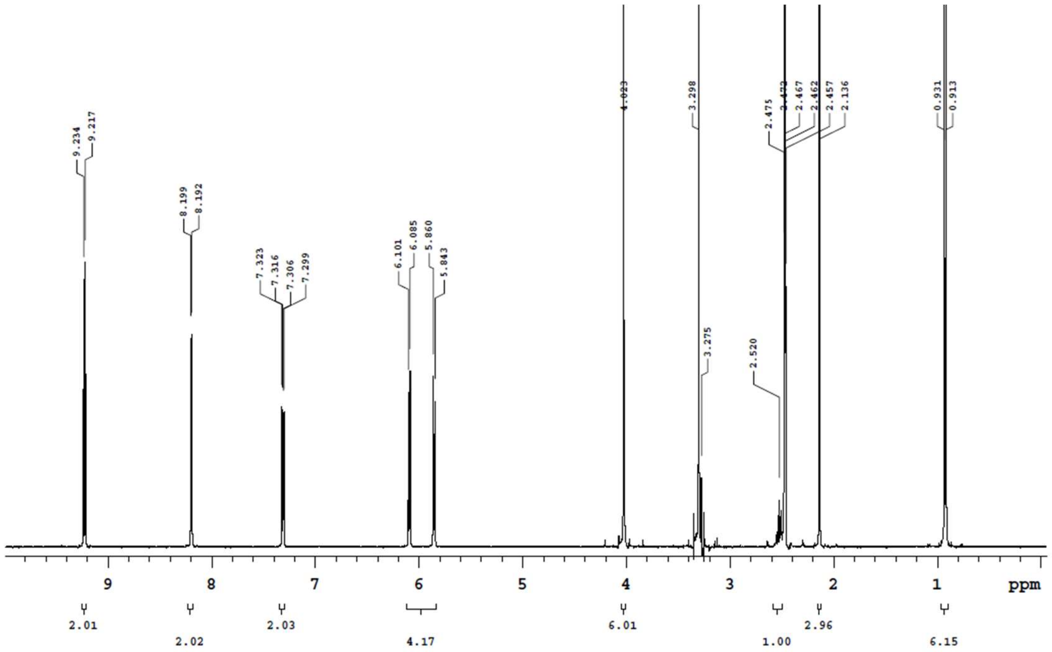

Figure S5. ^{1}H NMR spectrum of complex **RuBMeO** in DMSO-D_6.

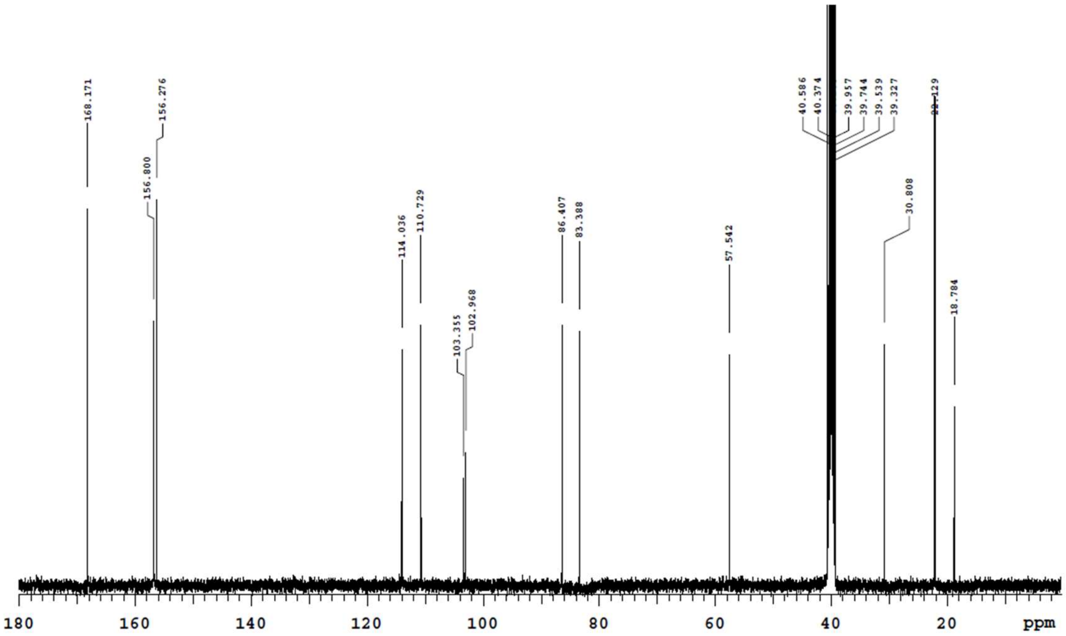

Figure S6. ^{13}C NMR spectrum of complex **RuBMeO** in DMSO-D_6.

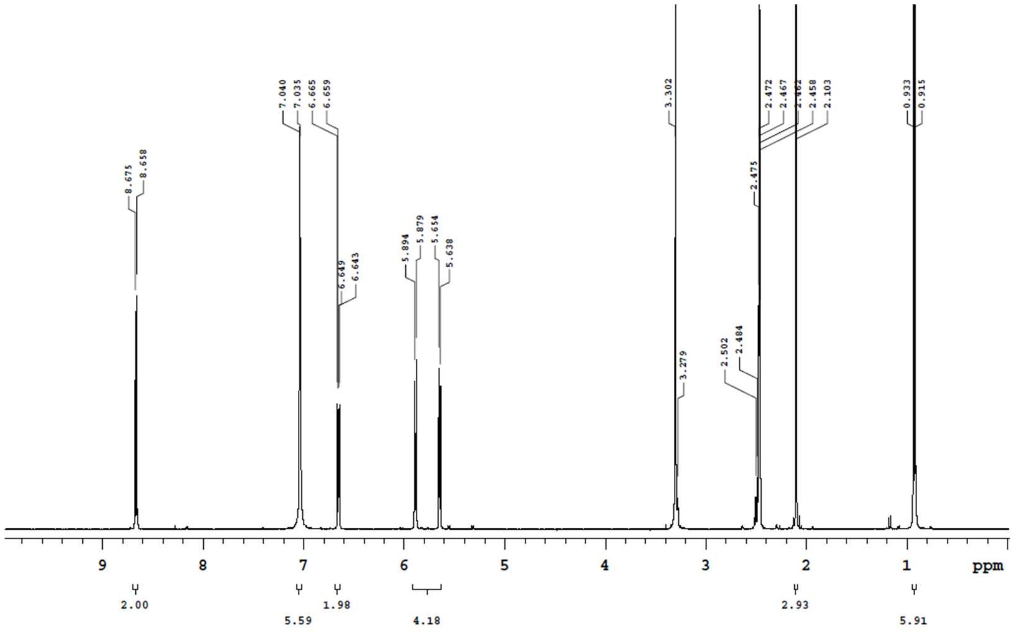

Figure S7. ^{1}H NMR spectrum of complex **RuBA** in DMSO-D$_6$.

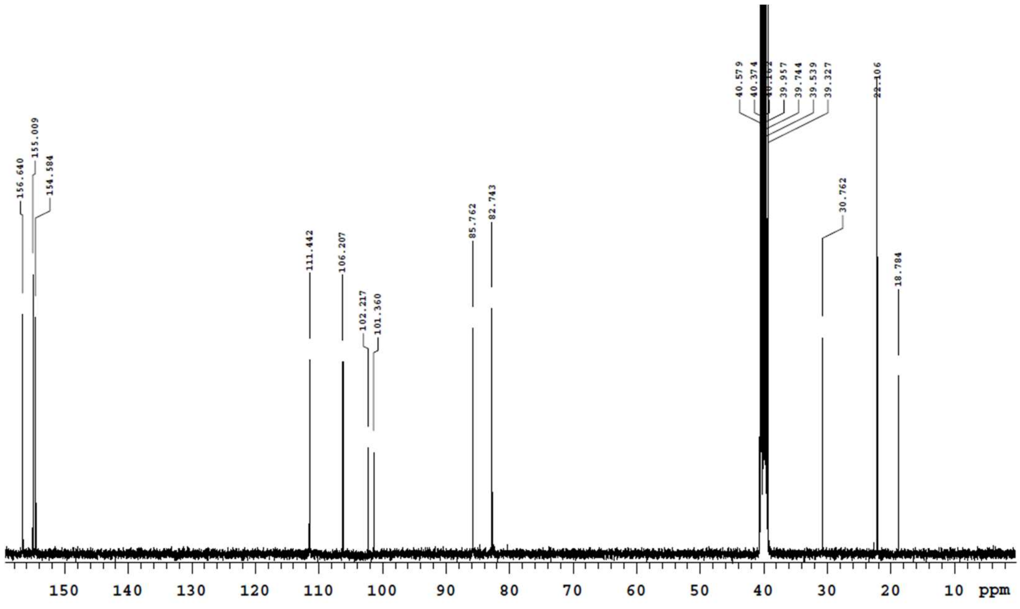

Figure S8. ^{13}C NMR spectrum of complex **RuBA** in DMSO-D$_6$.

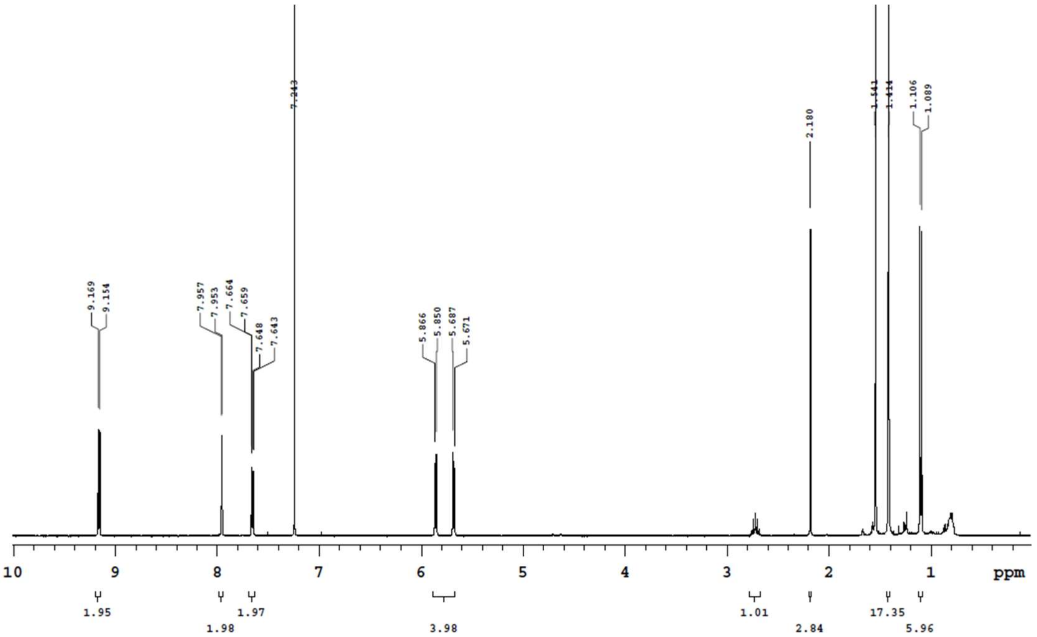

Figure S9. ^{1}H NMR spectrum of complex **RuBtB** in $CDCl_3$.

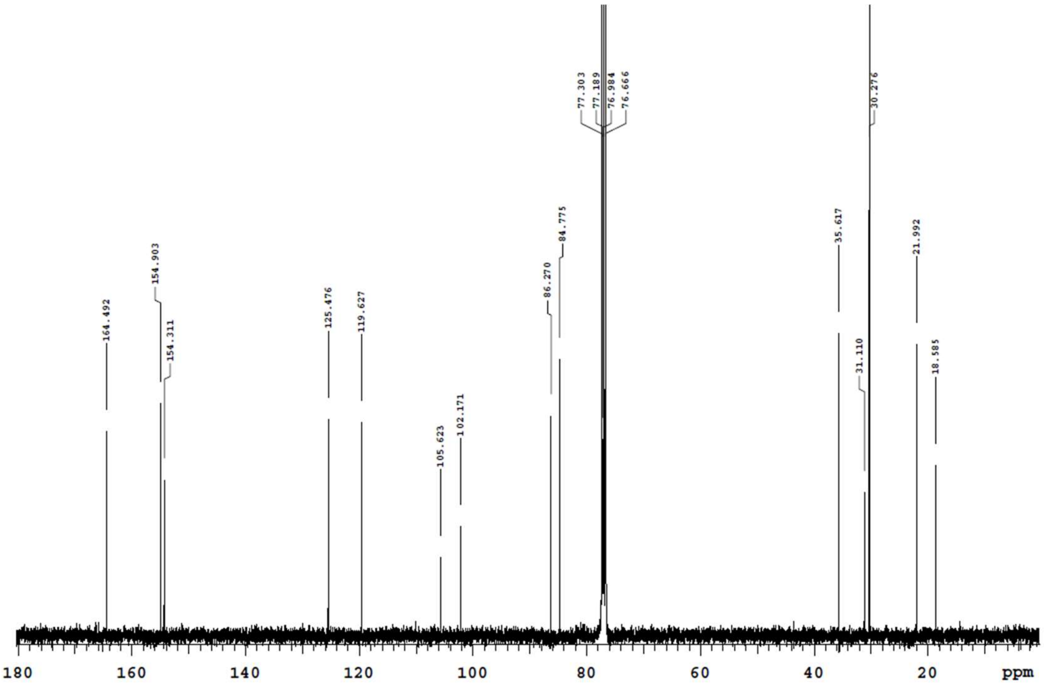

Figure S10. ^{13}C NMR spectrum of complex **RuBtB** in $CDCl_3$.

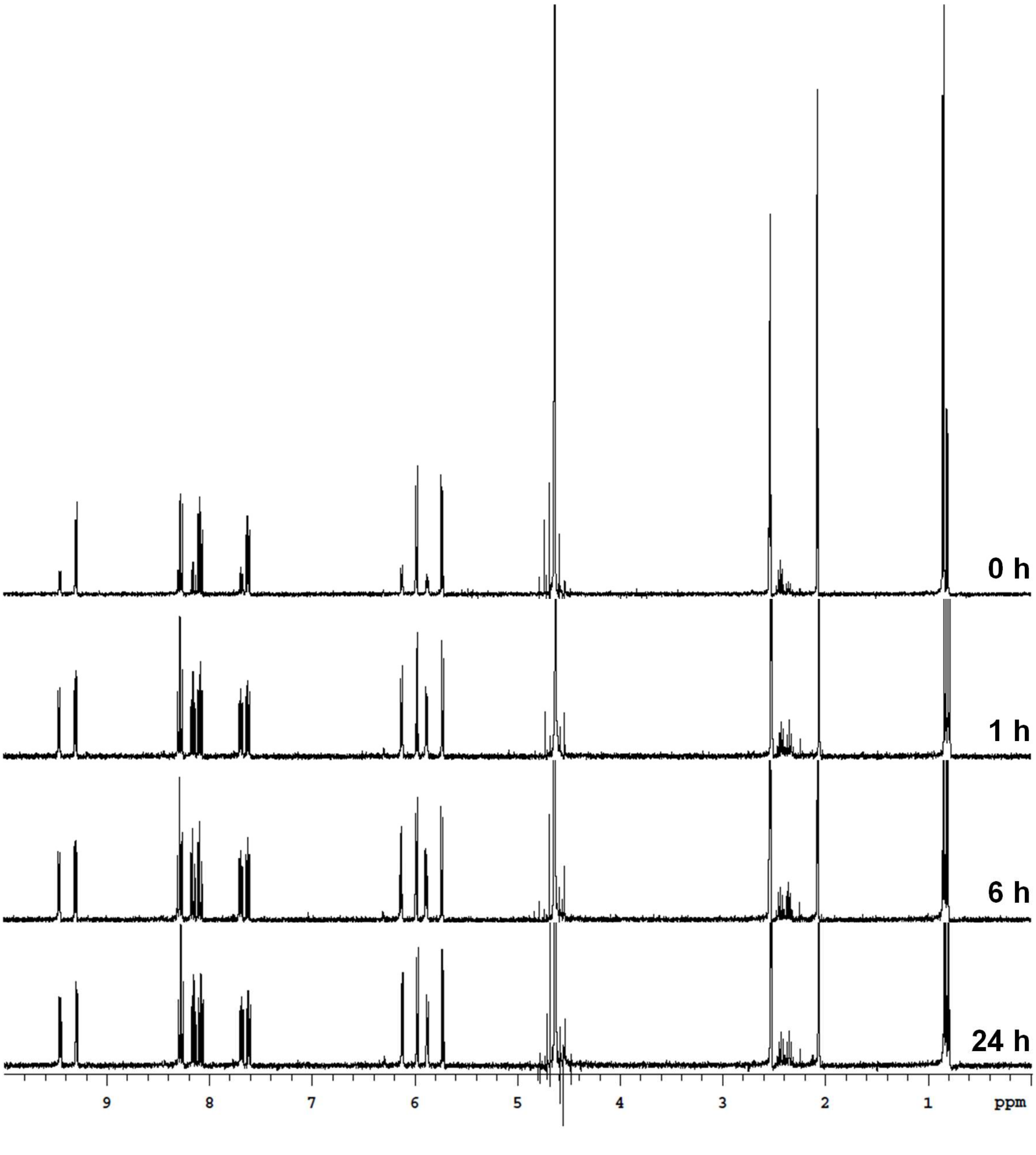

Figure S11. ^{1}H NMR spectrum of complex **RuB** in 25% DMSO-D_6 and D_2O over 24 hours.

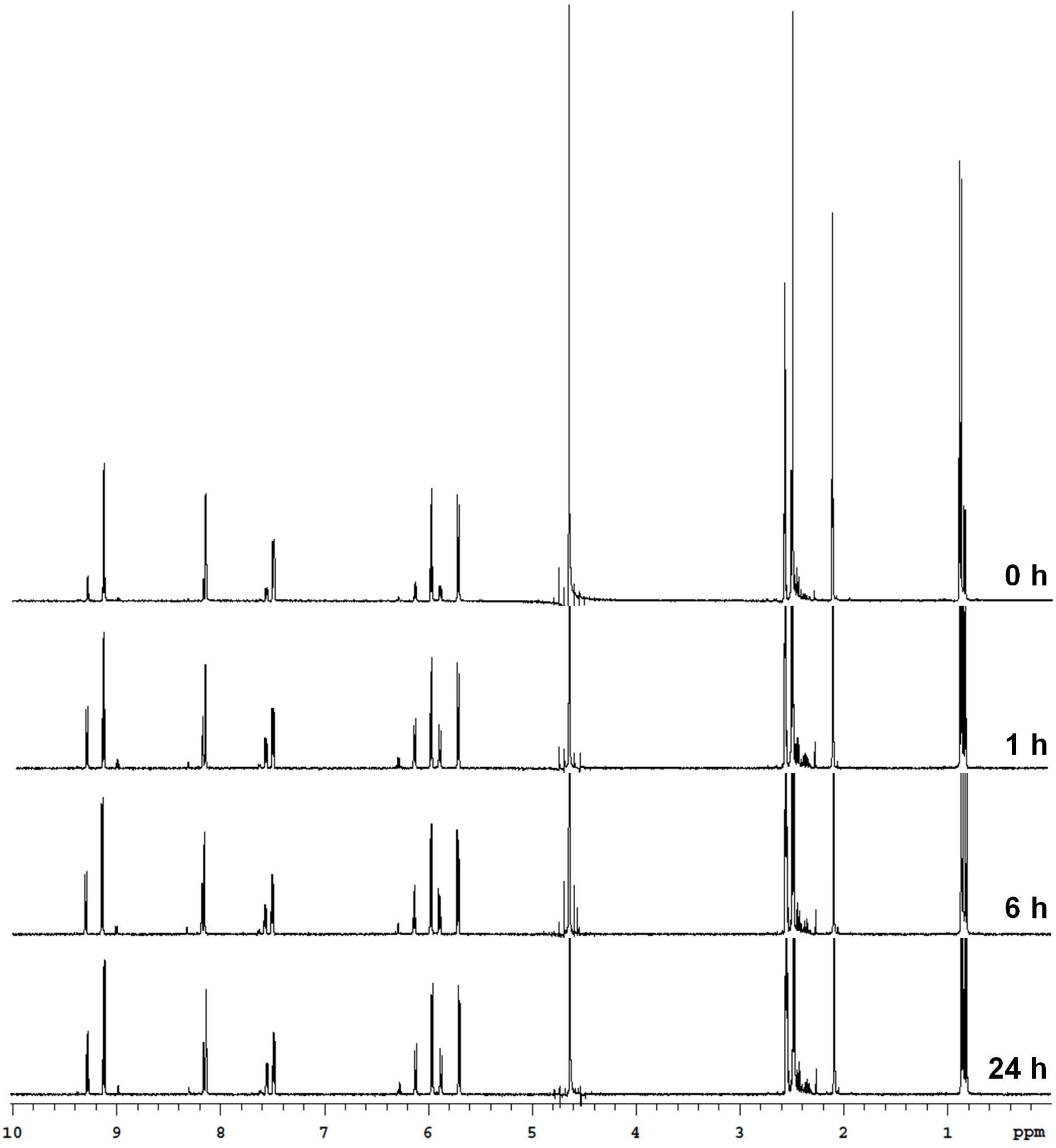

Figure S12. ^{1}H NMR spectrum of complex **RuBMe** in 25% DMSO-D_6 and D_2O over 24 hours.

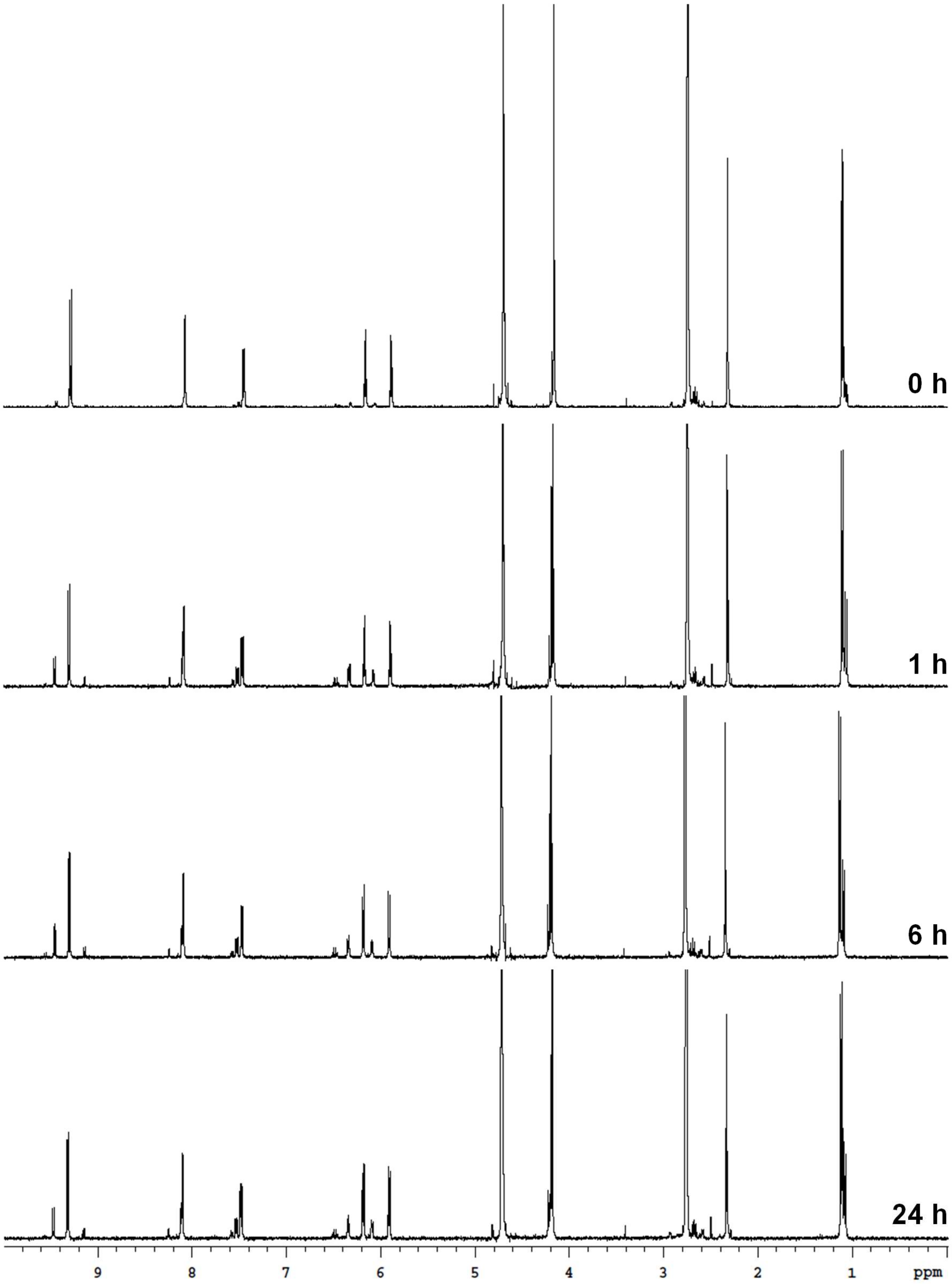

Figure S13. ^{1}H NMR spectrum of complex **RuBMeO** in 25% DMSO-D_6 and D_2O over 24 hours.

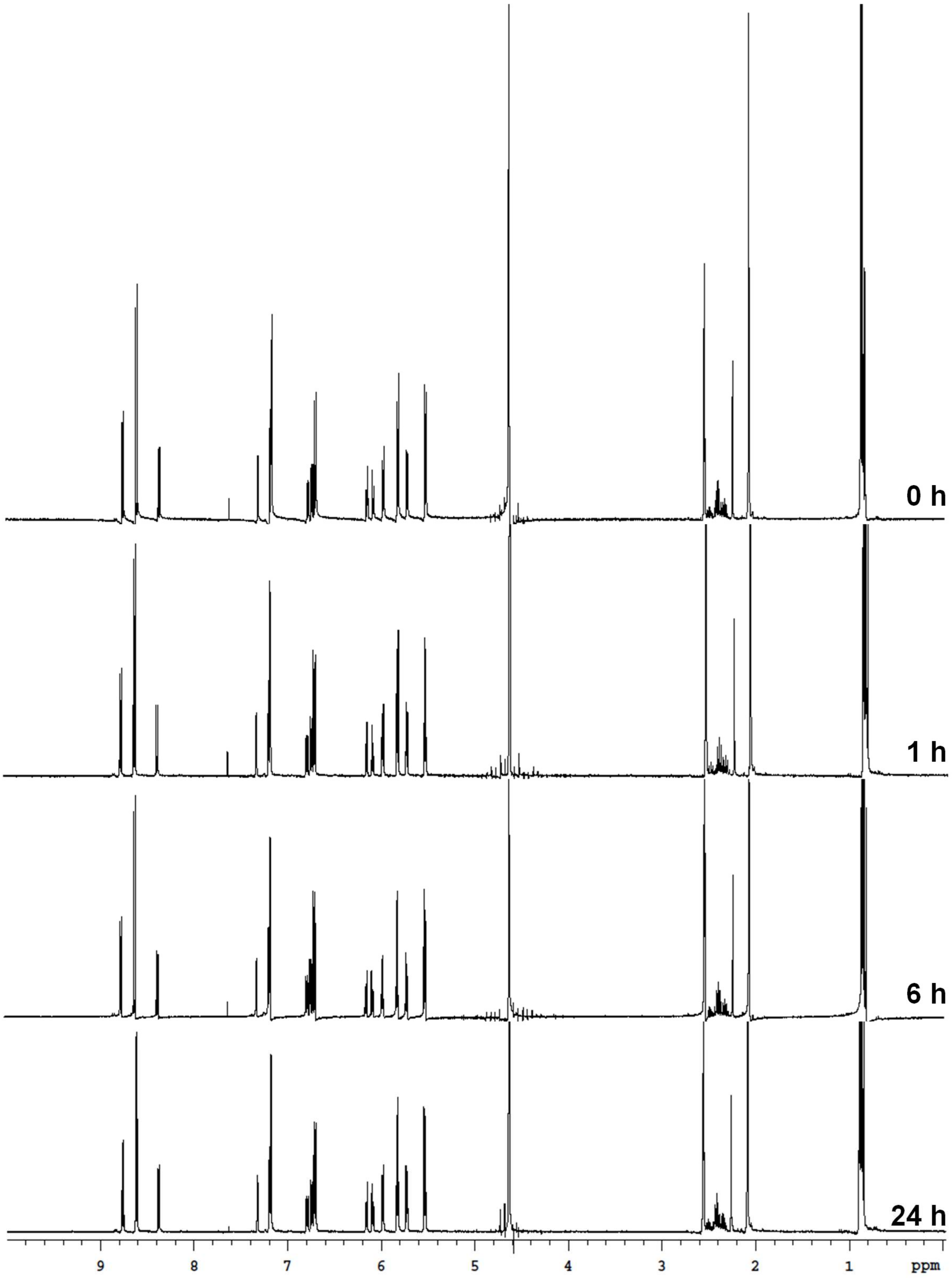

Figure S14. ^{1}H NMR spectrum of complex **RuBA** in 25% DMSO-D_6 and D_2O over 24 hours.

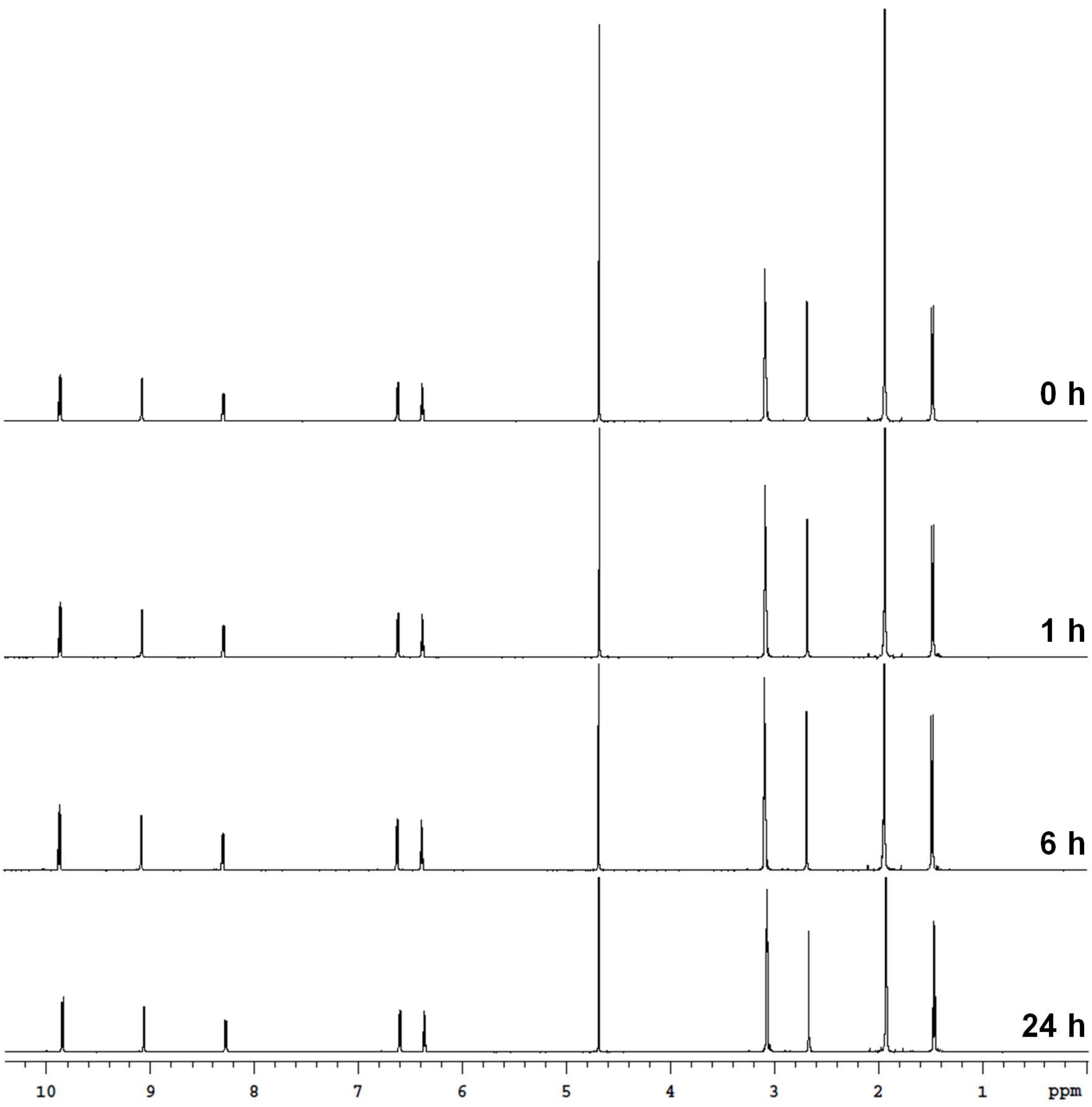

Figure S15. ^{1}H NMR spectrum of complex **RuBtB** in 75% DMSO-D_6 and D_2O over 24 hours.

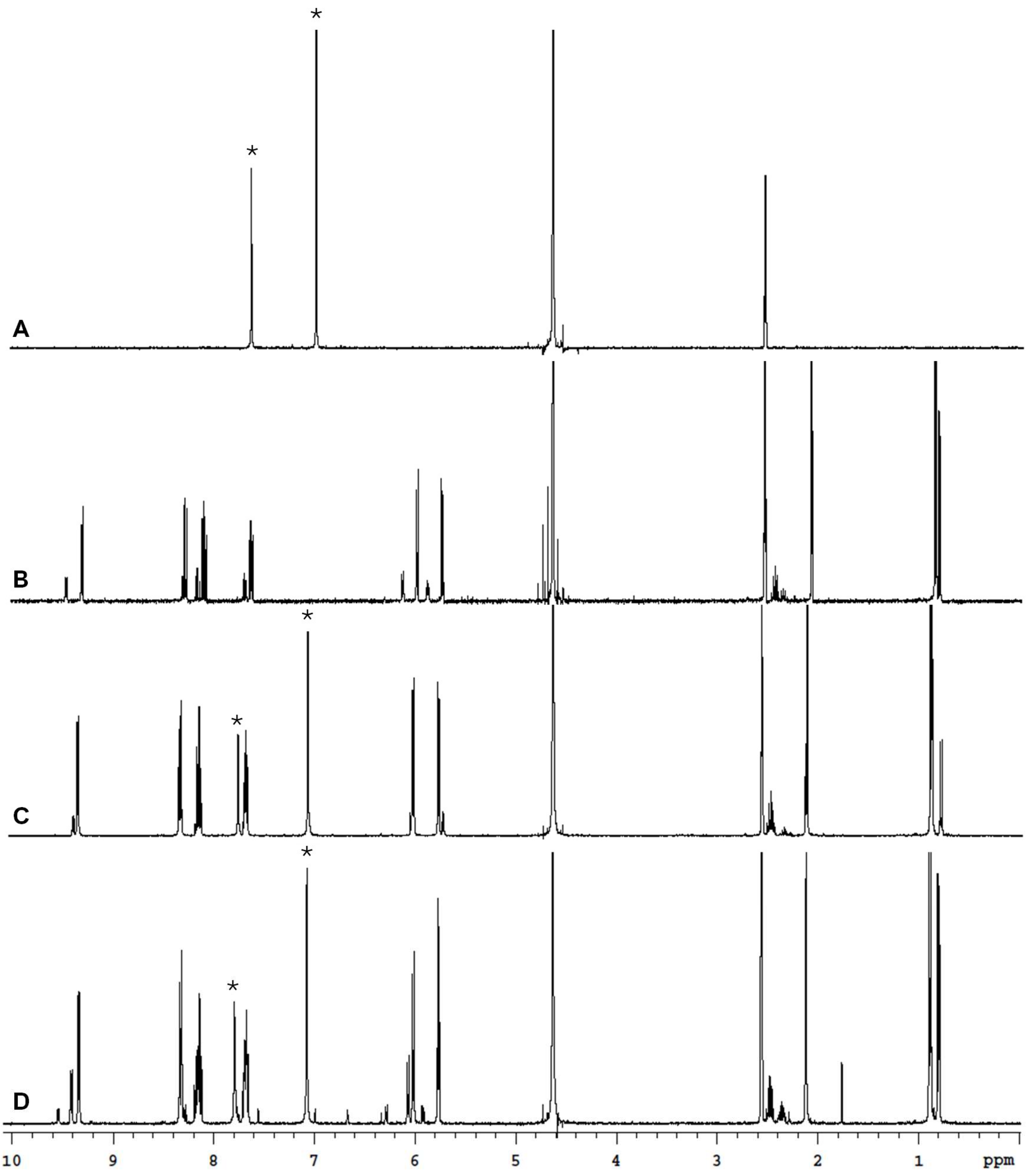

Figure S16. ^{1}H NMR spectra in 25% DMSO-D_6 and D_2O of complex **RuB** (18 µM) with imidazole (18 µM). A: Imidazole alone, B: **RuB** alone, C: Imidazole and **RuB** immediately after mixing, D: Imidazole and **RuB** after 1 hour of incubation at 37 °C. The free imidazole peaks are marked with a *.

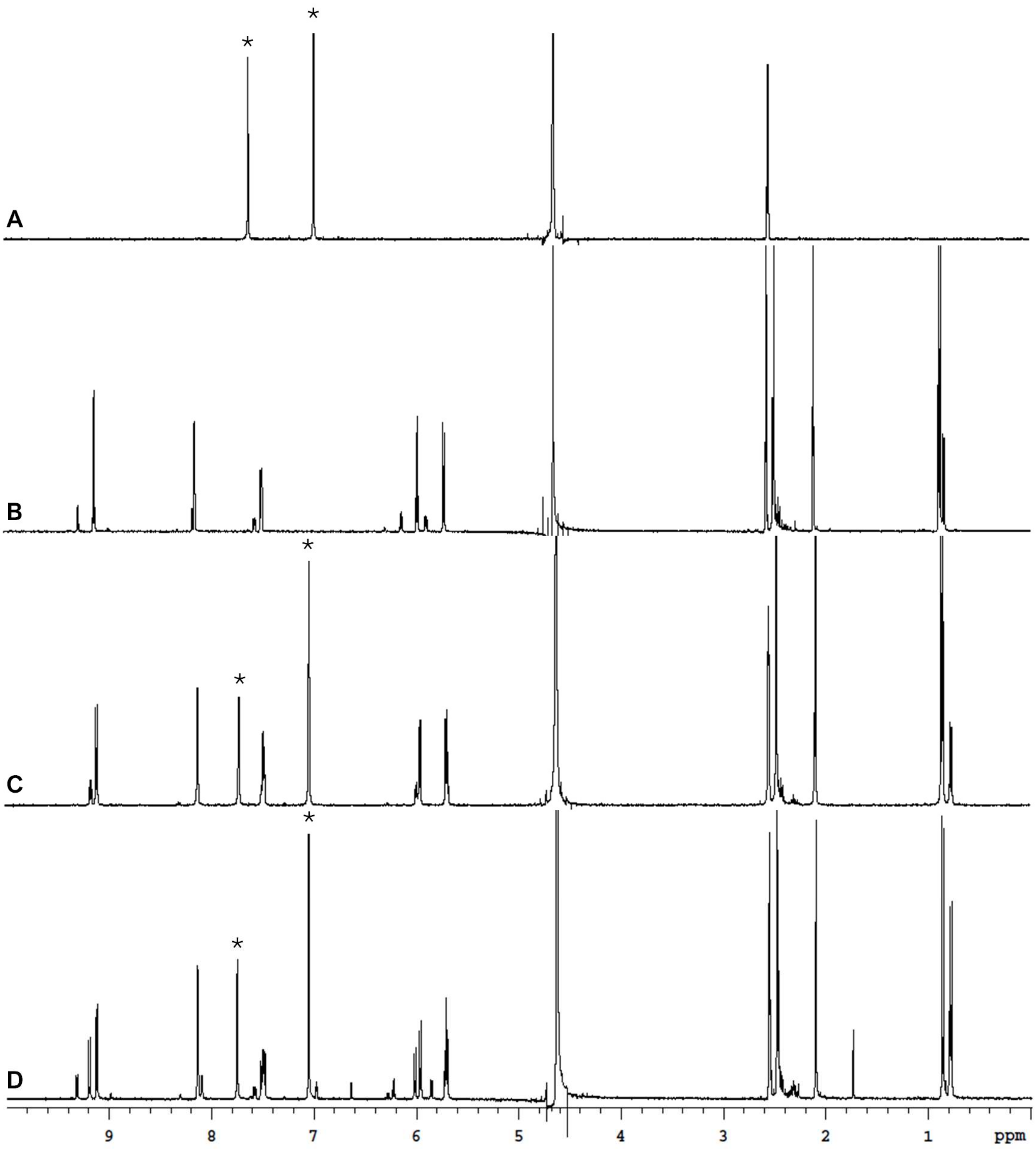

Figure S17. ^{1}H NMR spectra in 25% DMSO-D_6 and D_2O of complex **RuBMe** (18 μM) with imidazole (18 μM). A: Imidazole alone, B: **RuBMe** alone, C: Imidazole and **RuBMe** immediately after mixing, D: Imidazole and **RuBMe** after 1 hour of incubation at 37 °C. The free imidazole peaks are marked with a *.

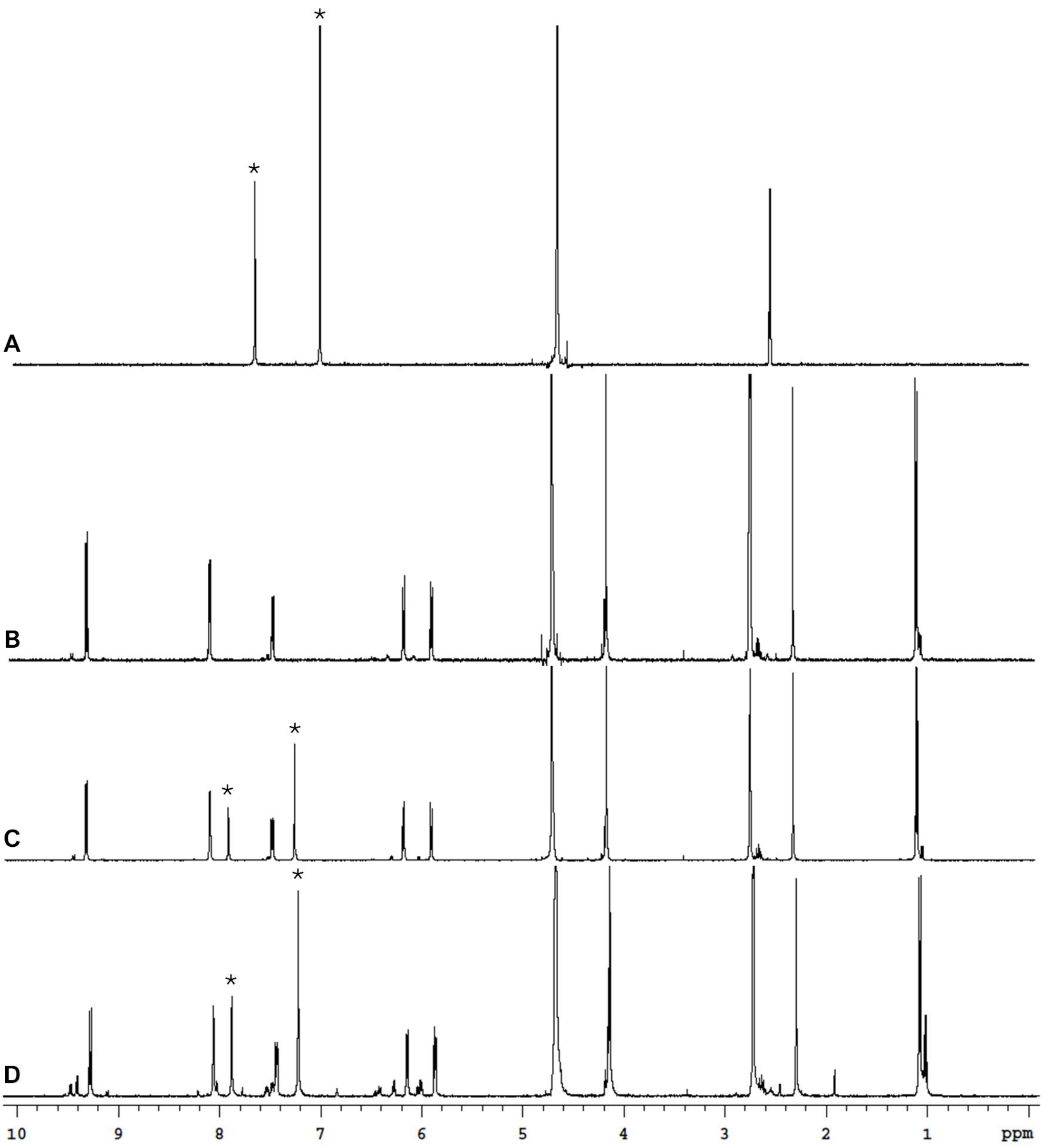

Figure S18. ^{1}H NMR spectra in 50% DMSO-D_6 and D_2O of complex **RuBMeO** (18 μM) with imidazole (18 μM). A: Imidazole alone, B: **RuBMeO** alone, C: Imidazole and **RuBMeO** immediately after mixing, D: Imidazole and **RuBMeO** after 1 hour of incubation at 37 °C. The free imidazole peaks are marked with a *.

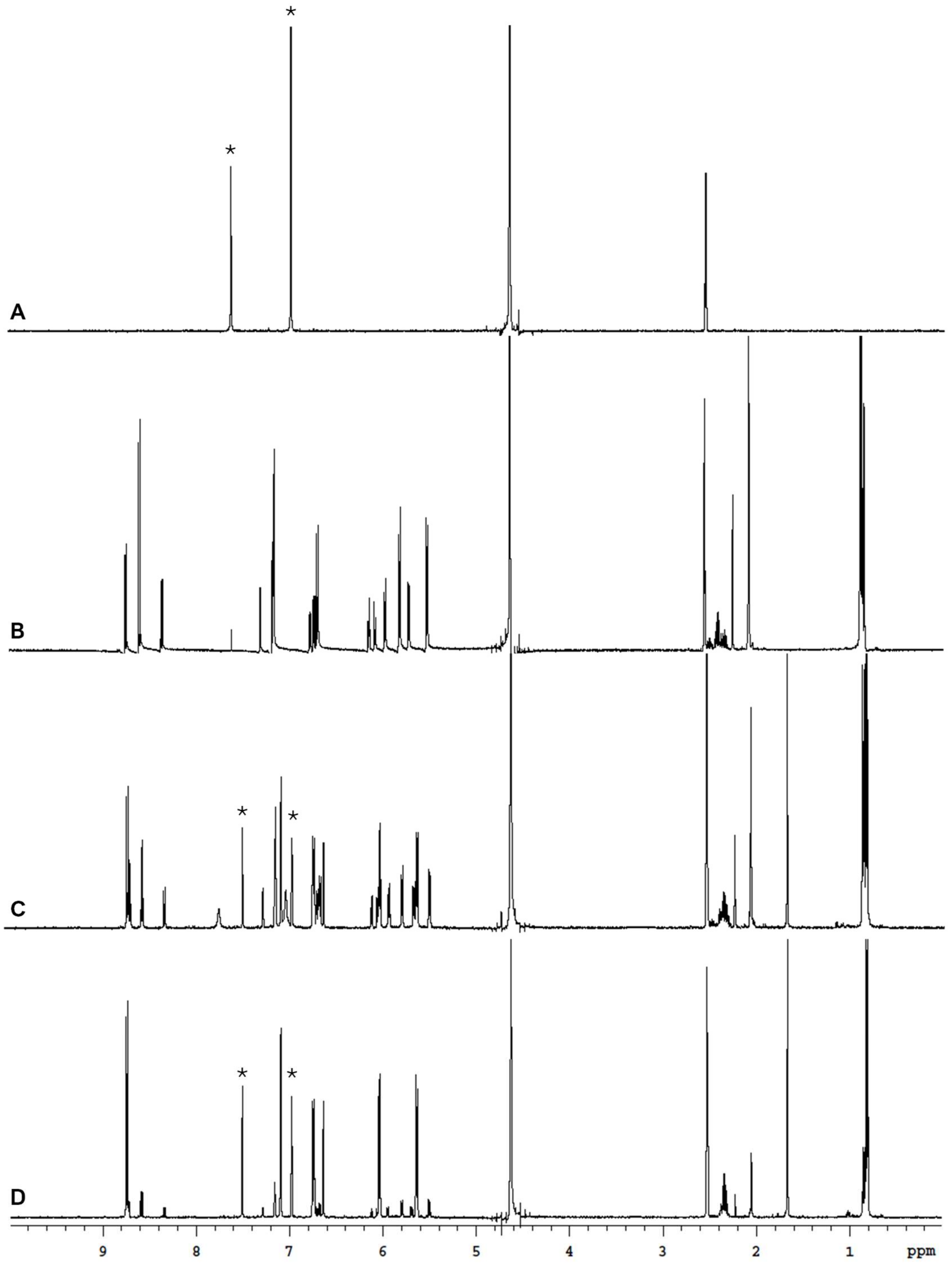

Figure S19. ^{1}H NMR spectra in 25% DMSO-D_6 and D_2O of complex **RuBA** (18 µM) with imidazole (18 µM). A: Imidazole alone, B: **RuBA** alone, C: Imidazole and **RuBA** immediately after mixing, D: Imidazole and **RuBA** after 1 hour of incubation at 37 °C. The free imidazole peaks are marked with a *.

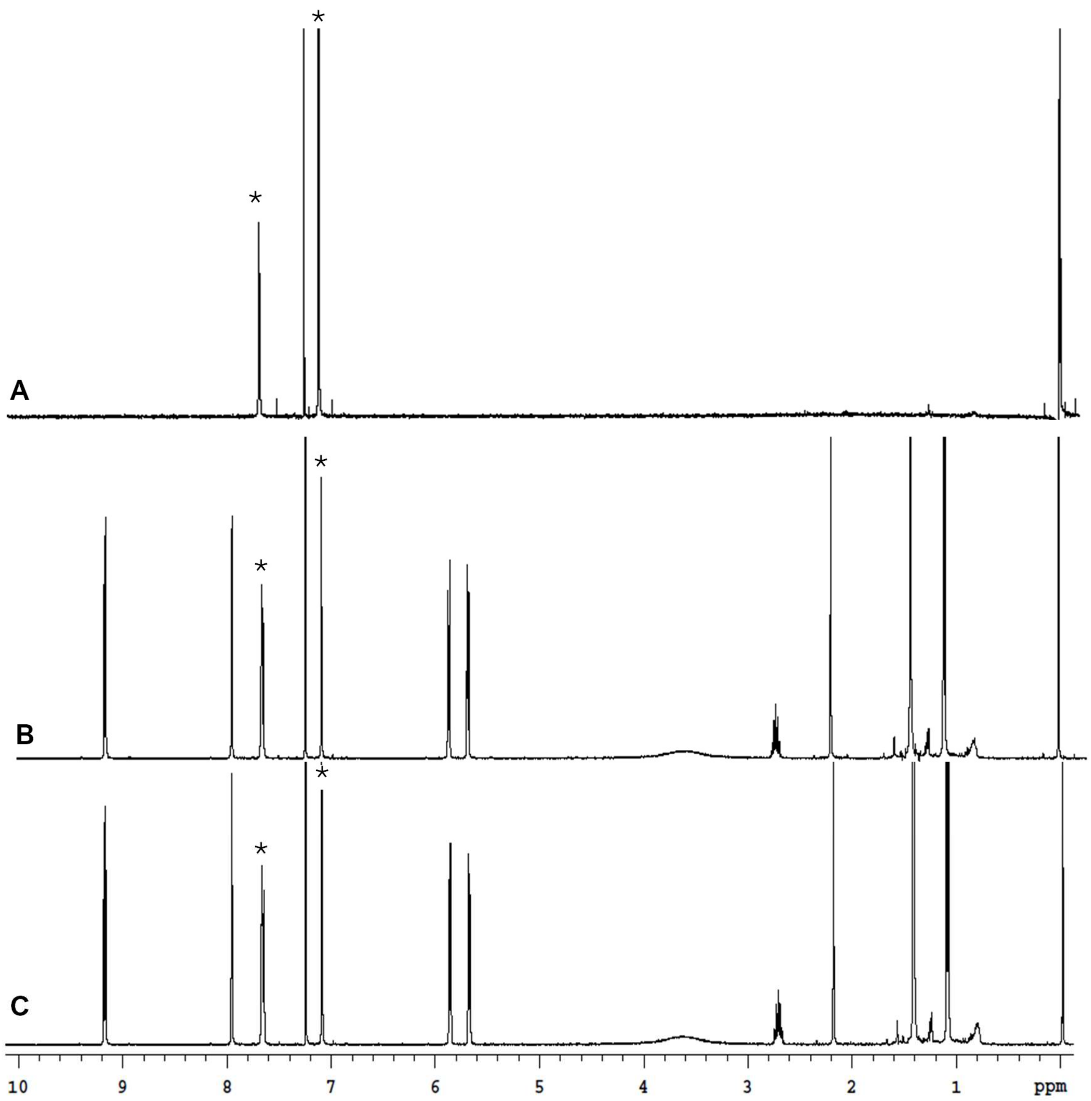

Figure S20. ^{1}H NMR spectra in $CDCl_3$ of complex **RuBtB** (18 μM) with imidazole (18 μM). A: Imidazole alone, B: Imidazole and **RuBtB** immediately after mixing C: Imidazole and **RuBtB** after 1 hour of incubation at 37 °C. The free imidazole peaks are marked with an asterisk (*).

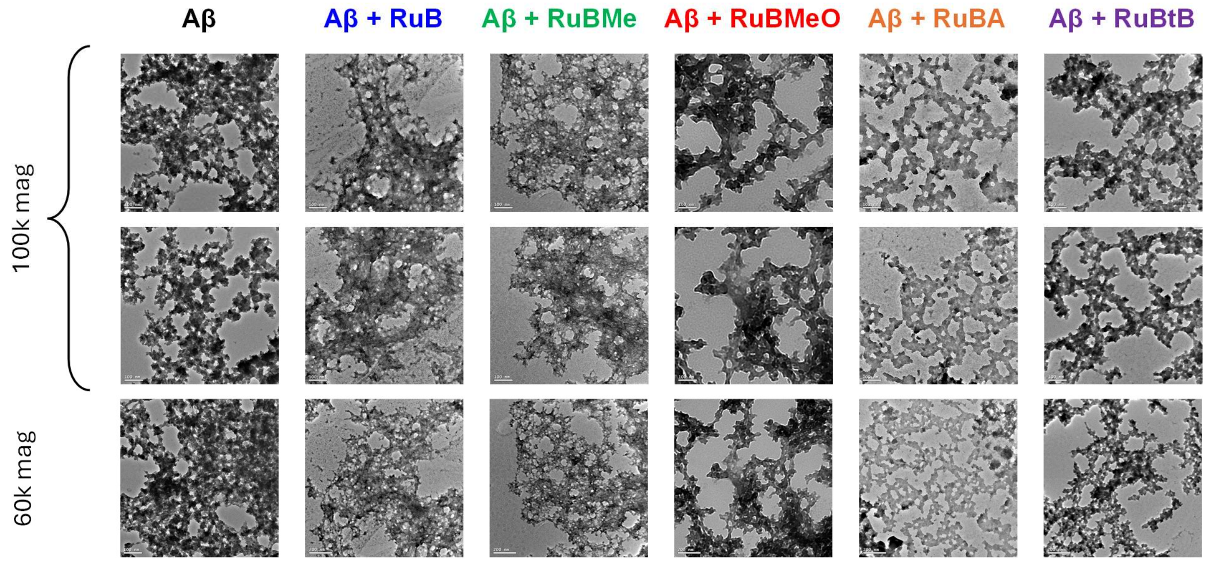

Figure S21. Additional TEM images collected for all of the Ru complexes with $A\beta_{40}$ from the DLS filtrates. Images in the top two rows were measured at 100 k magnification, while images in the bottom row was measured at 60 k magnification. Scale bars are provided within each image.

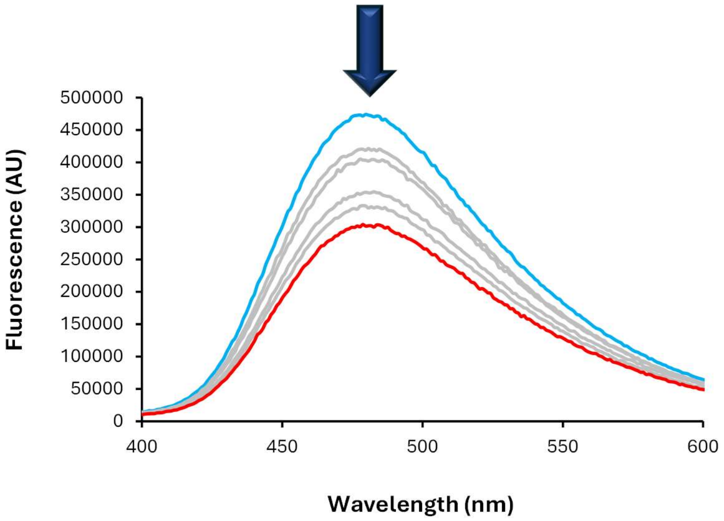

Figure S22. Fluorescence emission spectra at various Ru-HSA ratios by the titration of HSA-DG (1:1) with **RuB**. Experimental conditions: λ_{ex} = 330 nm, λ_{em} = 350-600 nm, [HSA] = [DG] = 2.5 μM, [Ru] = 0 (blue line), 12.5, 25, 32.5, 50, and 62.5 μM (red line), in PBS (pH 7.4).

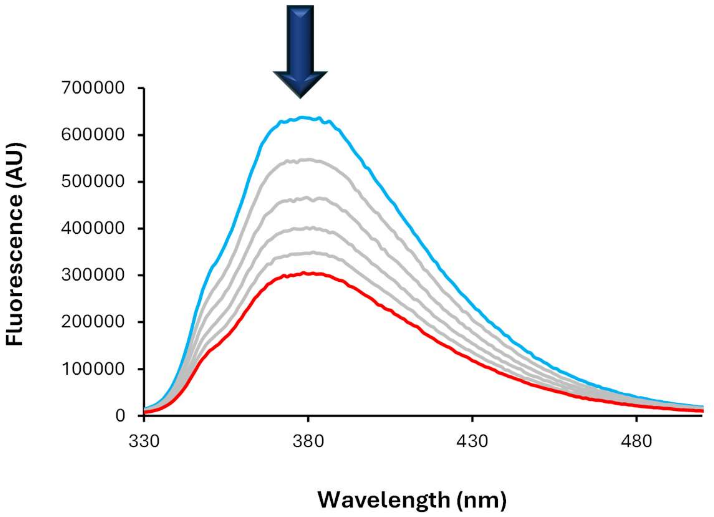

Figure S23. Fluorescence emission spectra at various Ru-HSA ratios by the titration of HSA-WF (1:1) with **RuB**. Experimental conditions: λ_{ex} = 295 nm, λ_{em} = 330-500 nm, [HSA] = [WF] = 2.5 μM, [Ru] = 0 (blue line), 5, 10, 15, 20, and 25 μM (red line), in PBS (pH 7.4).

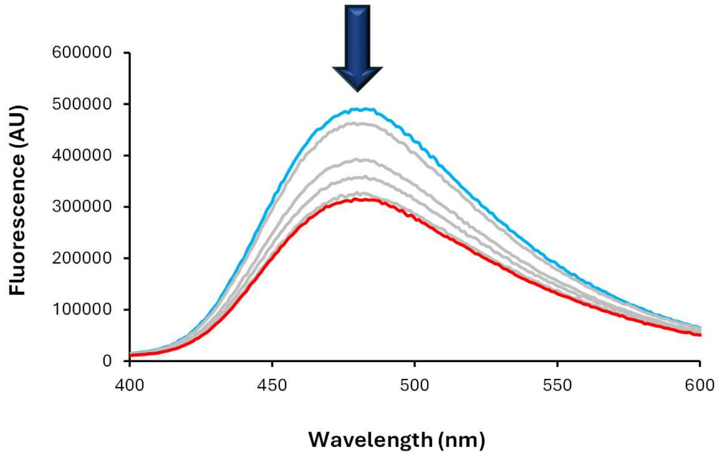

Figure S24. Fluorescence emission spectra at various Ru-HSA ratios by the titration of HSA-DG (1:1) with **RuBMe**. Experimental conditions: λ_{ex} = 330 nm, λ_{em} = 350-600 nm, [HSA] = [DG] = 2.5 μM, [Ru] = 0 (blue line), 12.5, 25, 32.5, 50, and 62.5 μM (red line), in PBS (pH 7.4).

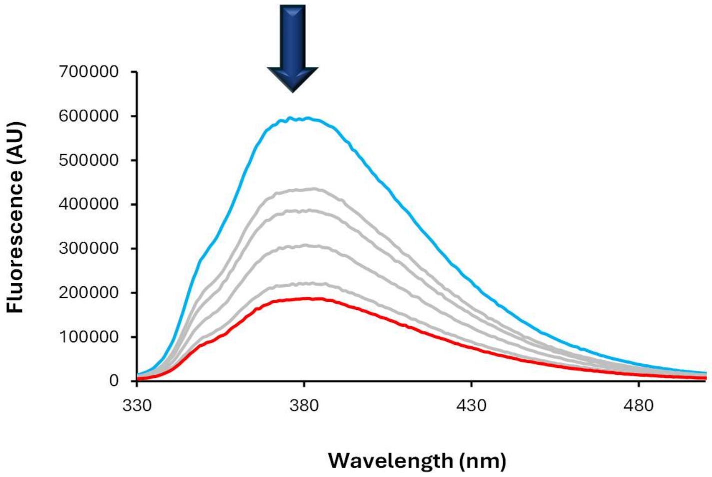

Figure S25. Fluorescence emission spectra at various Ru-HSA ratios by the titration of HSA-WF (1:1) with **RuBMe**. Experimental conditions: λ_{ex} = 295 nm, λ_{em} = 330-500 nm, [HSA] = [WF] = 2.5 μM, [Ru] = 0 (blue line), 12.5, 25, 32.5, 50, and 62.5 μM (red line), in PBS (pH 7.4).

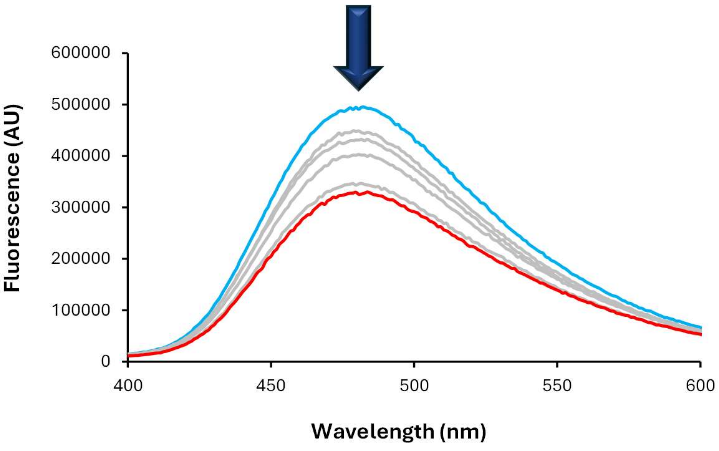

Figure S26. Fluorescence emission spectra at various Ru-HSA ratios by the titration of HSA-DG (1:1) with **RuBMeO**. Experimental conditions: λ_{ex} = 330 nm, λ_{em} = 350-600 nm, [HSA] = [DG] = 2.5 µM, [Ru] = 0 (blue line), 12.5, 25, 32.5, 50, and 62.5 µM (red line), in PBS (pH 7.4).

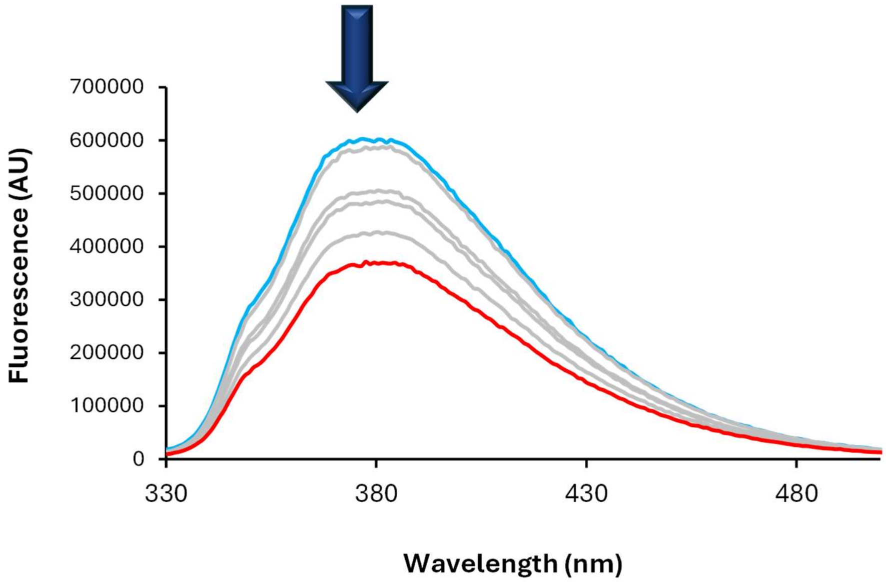

Figure S27. Fluorescence emission spectra at various Ru-HSA ratios by the titration of HSA-WF (1:1) with **RuBMeO**. Experimental conditions: λ_{ex} = 295 nm, λ_{em} = 330-500 nm, [HSA] = [WF] = 2.5 µM, [Ru] = 0 (blue line), 12.5, 25, 32.5, 50, and 62.5 µM (red line), in PBS (pH 7.4).

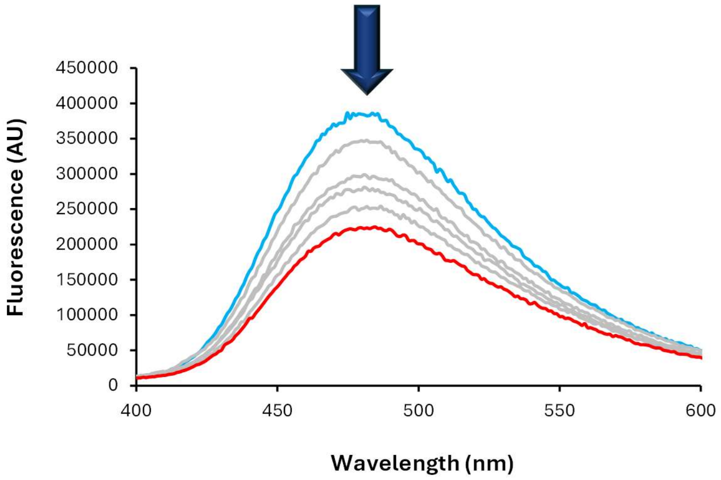

Figure S28. Fluorescence emission spectra at various Ru-HSA ratios by the titration of HSA-DG (1:1) with **RuBA**. Experimental conditions: λ_{ex} = 330 nm, λ_{em} = 350-600 nm, [HSA] = [DG] = 2.5 μM, [Ru] = 0 (blue line), 12.5, 25, 32.5, 50, and 62.5 μM (red line), in PBS (pH 7.4).

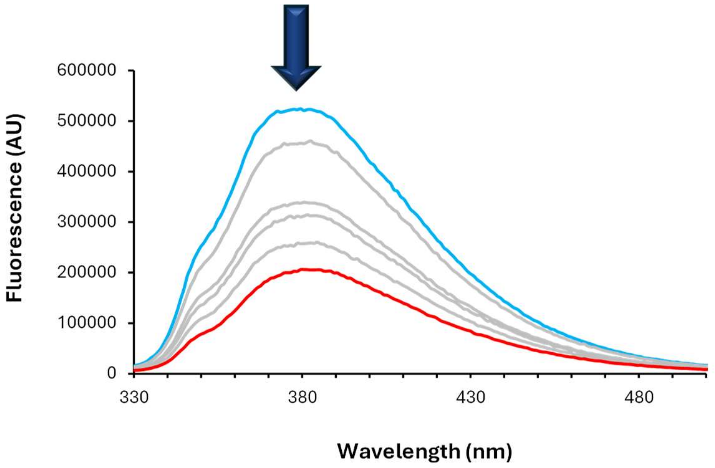

Figure S29. Fluorescence emission spectra at various Ru-HSA ratios by the titration of HSA-WF (1:1) with **RuBA**. Experimental conditions: λ_{ex} = 295 nm, λ_{em} = 330-500 nm, [HSA] = [WF] = 2.5 μM, [Ru] = 0 (blue line), 12.5, 25, 32.5, 50, and 62.5 μM (red line), in PBS (pH 7.4).

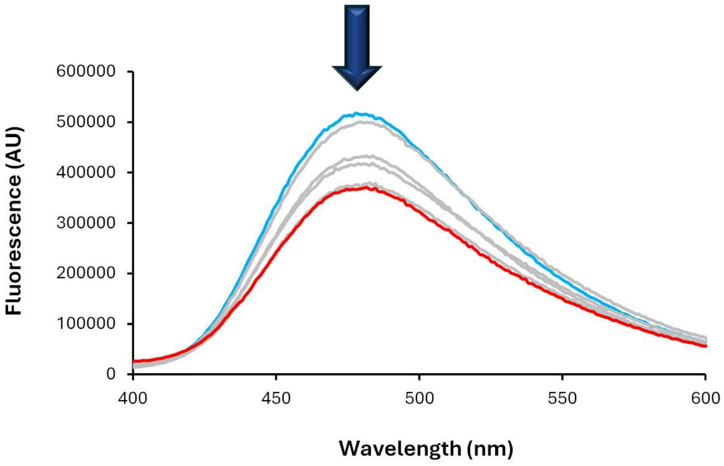

Figure S30. Fluorescence emission spectra at various Ru-HSA ratios by the titration of HSA-DG (1:1) with **RuBtB**. Experimental conditions: λ_{ex} = 330 nm, λ_{em} = 350-600 nm, [HSA] = [DG] = 2.5 µM, [Ru] = 0 (blue line), 12.5, 25, 32.5, 50, and 62.5 µM (red line), in PBS (pH 7.4).

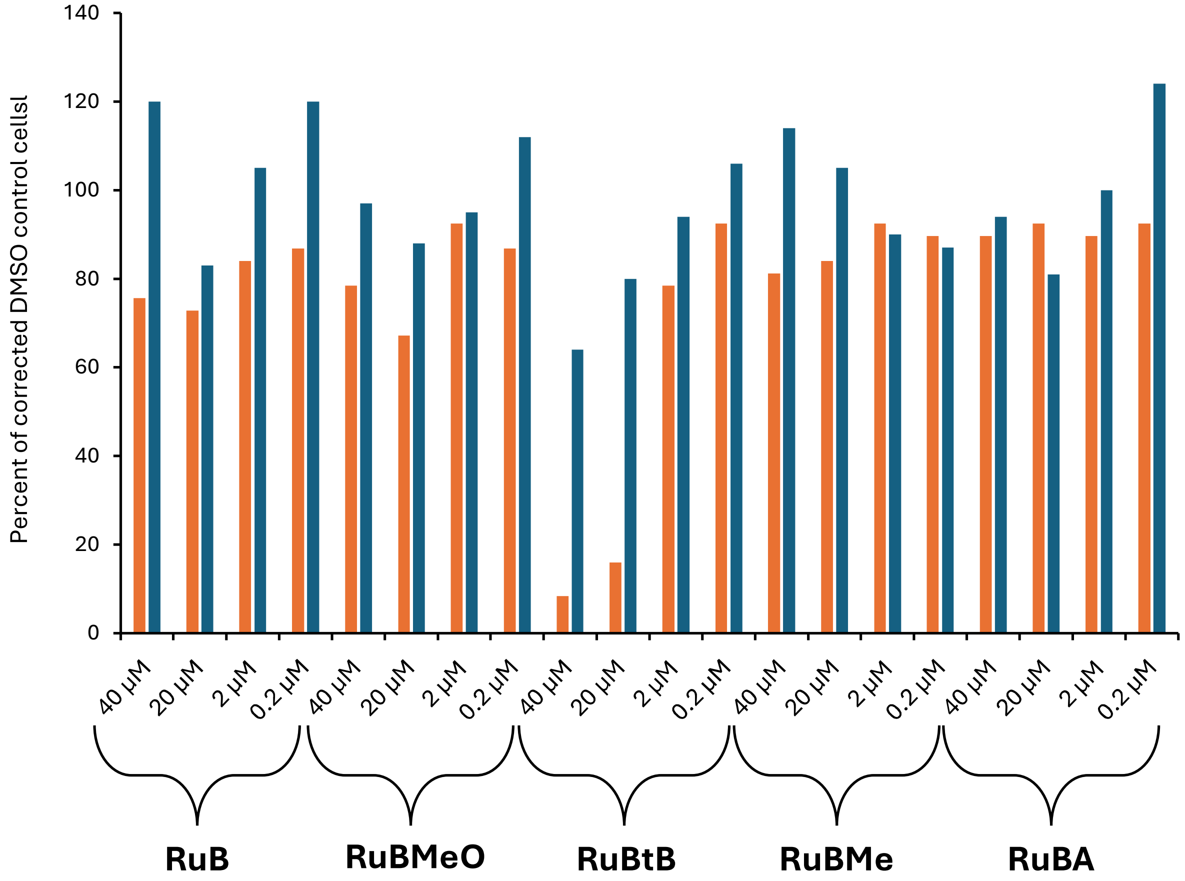

Figure 31. Cell viability for C6 (orange) and PC12 (blue) cells, as determined by MTT following incubation with the respective Ru complexes.

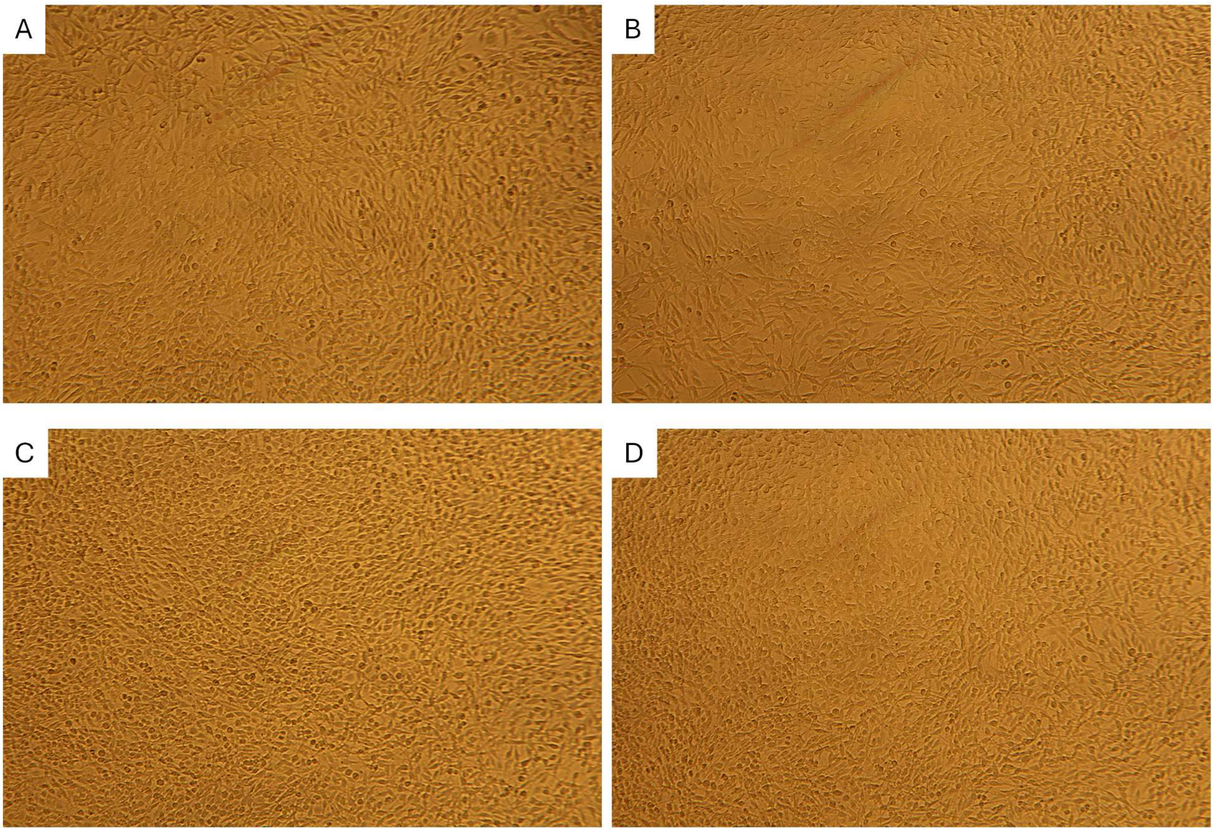

Figure S32. Confocal images of the C6 cells following 24 hours of incubation with A) DMSO, B) 20 μM **RuB**, C) 20 μM **RuBMe**, and D) 20 μM **RuBMeO**.

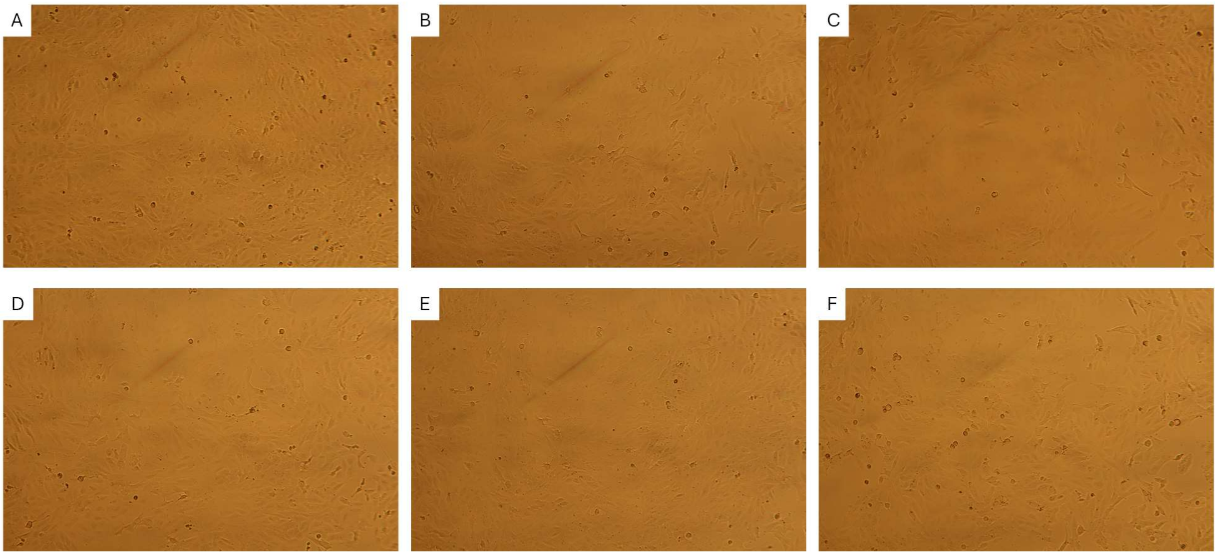

Figure S33. Confocal images of the P12 cells following 24 hours of incubation with A) DMSO, B) 20 μM **RuB**, C) 20 μM **RuBMe**, and D) 20 μM **RuBMeO**, E) 20 μM **RuBA**, and F) 20 μM **RuBtB**.

Table S1. Crystal data and structure refinement for **RuBA**.

CCDC Number	2420394	
Empirical formula	C20 H24 Cl F6 N4 P Ru	
Formula weight	601.92	
Temperature	100.00(10) K	
Wavelength	1.54184 Å	
Crystal system	triclinic	
Space group	*P-1*	
Unit cell dimensions	*a = 11.16430(10) Å*	*a = 91.9690(10)°*
	b = 13.77240(10) Å	*b = 109.3060(10)°*
	c = 16.7407(2) Å	*g = 94.0570(10)°*
Volume	2418.64(4) $Å^3$	
Z	4	
Density (calculated)	1.653 Mg/m^3	
Absorption coefficient	7.461 mm^{-1}	
F(000)	1208	
Crystal color, morphology	yellow, block	
Crystal size	0.156 x 0.093 x 0.044 mm^3	
Theta range for data collection	2.802 to 80.223°	
Index ranges	$-13 \leq h \leq 14$, $-17 \leq k \leq 17$, $-21 \leq l \leq 20$	
Reflections collected	50384	
Independent reflections	10347 [*R*(int) = 0.0396]	
Observed reflections	9510	
Completeness to theta = 74.504°	99.6%	
Absorption correction	Multi-scan	
Max. and min. transmission	1.00000 and 0.89034	
Refinement method	Full-matrix least-squares on F^2	
Data / restraints / parameters	10347 / 560 / 745	
Goodness-of-fit on F^2	1.034	
Final *R* indices [*I*>2sigma(*I*)]	*R*1 = 0.0406, *wR*2 = 0.0973	
R indices (all data)	*R*1 = 0.0440, *wR*2 = 0.0994	
Largest diff. peak and hole	1.005 and -1.146 e.$Å^{-3}$	

Table S2. Hydrogen bonds and close contacts for **RuBA** [Å and °].

D-H...A	d(D-H)	d(H...A)	d(D...A)	<(DHA)
N7-H7A...F10#1	0.88	2.32	3.137(10)	154.9
N7-H7A...F8A#1	0.88	2.46	3.158(11)	136.2
N8-H8D...F9#2	0.88	2.07	2.887(8)	154.0
N4-H4B...F11A#3	0.89(5)	2.36(5)	3.174(9)	152(4)

Symmetry transformations used to generate equivalent atoms:
#1 -x+2,-y+2,-z+1 #2 x-1,y,z #3 -x+1,-y+1,-z
